# Approximations of Cumulants of the Stochastic Power Law Logistic Model

**DOI:** 10.1007/s11538-019-00687-w

**Published:** 2020-01-22

**Authors:** Ingemar Nåsell

**Affiliations:** Department of Mathematics, The Royal Institute of Technology, 100 44 Stockholm, Sweden

**Keywords:** Asymptotic approximations, Quasi-stationary distributions, Logistic model

## Abstract

Asymptotic approximations of the first three cumulants of the quasi-stationary distribution of the stochastic power law logistic model are derived. The results are based on a system of ODEs for the first three cumulants. We deviate from the classical moment closure approach by determining approximations without closing the system of equations. The approximations are explicit in the model’s parameters, conditions for validity of the approximations are given, magnitudes of approximation errors are given, and spurious solutions are easily detected and eliminated. In these ways, we provide improvements on previous results for this model.

## Introduction

We study a stochastic version of the power law logistic model. Logistic models are used as models for the size of a population with density-dependent growth. This means that the net growth rate per individual is a decreasing function of the population size. The simplest decreasing function is the linear one. It leads to the classical logistic model, whose deterministic version was studied by Verhulst already in 1838. The stochastic version of this model has been studied extensively, as shown by Nåsell (2017). More general deterministic power law logistic models have been discussed by Banks (1994) and by Tsoularis and Wallace (2002), while stochastic versions of such models are treated by Matis et al. (1998), by Renshaw (2011), and by Bhowmick et al. (2016).

The model that we deal with is a Markov Chain with finite state space and continuous time, and with an absorbing state at the origin. It can also be described as a finite-state birth-death process. Two model formulations are given in Sect. 2. They are mathematically equivalent, but with different parameters. The maximum population size *N* is an important parameter in the second one of the two formulations. This formulation is basic for our work, since *N* serves the role of being the large parameter for which asymptotic approximations of various quantities can be derived.

The state variable *X*(*t*) of either of the two formulations is interpreted as the number of individuals in a population. Ultimate extinction of this variable is a fact for this model. This means in particular that the stationary distribution of *X*(*t*) is degenerate with probability one at the origin. A related and important random variable $X^Q(t)$ is defined by conditioning *X*(*t*) on non-extinction. The stationary distribution of this conditioned random variable is the so-called quasi-stationary distribution (QSD). The QSD is important for the information it gives about the long-term behavior of a surviving population. Our main goal is to derive approximations of the first three cumulants of the QSD.

Our approach toward this goal resembles the well-known moment closure method in the sense that both are based on a system of ODEs for the first few (three) cumulants. This system of ODEs is not closed: the number of unknown functions (cumulants) is larger than the number of equations. This fact has caused all practitioners of moment closure to introduce an approximating assumption that leads to closure of the system of ODEs. The method is used widely, as shown by the recent review paper by Kuehn (2016). However, it is also known to possess several weaknesses. The first of these weaknesses is that no condition for validity of the resulting approximation is available, a second one is that the magnitude of the approximation error can not be evaluated, a third one is that it often leads to spurious solutions that require large efforts to eliminate, and a fourth one is that the dependence of the approximating expressions on the model’s parameters is not known. An early basic paper in the area was written by Whittle (1957). The desirability of closing the system of ODEs for the cumulants cannot be denied. It is indeed a necessity if the goal is to derive exact expressions for the cumulants. However, it is important to realize that closure is not necessary if one is satisfied with approximate results, as we are in this paper.

We do not use moment closure methods at all. Instead, we apply the alternative mathematical method introduced in Nåsell (2017). Thus, we base our work on a system of ODEs for cumulants, just as is done by practitioners of moment closure. However, we avoid the step that leads to closure of the system of ODEs, since the ad hoc nature of this step is the cause of several weaknesses of the moment closure method. Our alternative is to search for asymptotic approximations. This requires the reparameterization that accompanies the second one of the two model formulations of Sect. 2. To proceed with our approach, we also need information about the orders of magnitude (in terms of *N*) of the cumulants that appear in the system of ODEs. We motivate our assumptions in this regard with numerical evaluations. The method that we use avoids the weaknesses associated with moment closure methods.

Methods for numerical evaluations of the QSD and of the associated cumulants are discussed in Sect. 3. Our approach is different from what has been used by previous workers on this model. Results of numerical evaluations are used to motivate forms of basic assumptions that are made for determining asymptotic approximations of the first few cumulants of the QSD and also for checking the orders of magnitude of the errors of the approximations that we derive.

Sections 4 and 5 study the two random variables *X*(*t*) and $X^Q(t)$, respectively. We give a system of first-order ODEs for the first 3 cumulants of the unconditioned random variable *X*(*t*) in Sect. 4. The same system of equations is shown in Sect. 5 to provide an approximation for the system of ODEs of the first 3 cumulants of the conditioned random variable $X^Q(t)$ in a particular parameter region. This means that approximations of the cumulants of the QSD in this region are found as critical points of the system of ODEs. Asymptotic approximations of the coordinates of the critical points of this system of ODEs are derived in Sect. 6. They serve to give asymptotic approximations of the first 3 cumulants of the QSD. These results hold for small positive integer values of the parameter *s* that describes the power of the population size *n* that gives the decreasing function of the population size that reflects the density dependence of the net birth rate of the model. An extension of these results to arbitrary positive values of *s*, both integer and non-integer, is proposed in Sect. 7. Comparisons are made in Sect. 8 with published results that also deal with the cumulants of the QSD of the same model. The paper ends with some concluding comments in Sect. 9.

## Model Formulation

The stochastic power law logistic model is a model for the size of a population with density-dependent growth. It is formulated as a birth-death process $\{X(t),t\ge 0\}$. The hypotheses of the model are summarized in the descriptions of the population birth rate $\lambda _n$ and the population death rate $\mu _n$ as functions of the state *n* of the process. The formulation given by Matis et al. (1998) takes the following form: the population birth rate equals

1 $$\begin{aligned} \lambda _n = {\left\{ \begin{array}{ll} (a_1 - b_1 n^s) n, &{}\quad n\le (a_1/b_1)^{1/s}, \\ 0, &{}\quad \text {otherwise}, \end{array}\right. } \end{aligned}$$

and the population death rate is

2 $$\begin{aligned} \mu _n = (a_2 + b_2 n^s)n. \end{aligned}$$

The state space appears to be unbounded and equal to $\{0,1,2,\dots \}$. The parameters $a_1, b_1, a_2, b_2, s$ are all assumed to be positive. This model formulation takes its form from the similar model introduced by Bartlett (1957) for the special case of the Verhulst logistic model where $s=1$.

We shall use a different formulation, where

3 $$\begin{aligned}&\lambda _n = \mu R_0 \left( 1 - \left( \frac{n}{N}\right) ^s \right) n, \quad n=0,1,\dots ,N, \end{aligned}$$

4 $$\begin{aligned}&\mu _n = \mu \left( 1 + \alpha \left( \frac{n}{N}\right) ^s \right) n, \quad n=0,1,\dots ,N. \end{aligned}$$

The state space of the process in this formulation is finite and equal to $\{0,1,2,\dots ,N\}$. The parameter space contains the five parameters $N, R_0, \alpha , \mu$, and *s*. Among these, *N* is a large positive integer that represents the maximum population size, $R_0$ is a positive dimensionless threshold parameter, $\alpha$ is a nonnegative dimensionless parameter, $\mu$ is a positive death rate with dimension inverse time, and *s* is a positive dimensionless number. This model formulation is an extension from the formulation of the logistic Verhulst model, corresponding to $s=1$, given by Nåsell (2017). An advantage of this formulation over the previous one is that dimensions and biological interpretations of the parameters are known.

Under the assumption that the initial distribution is supported on the state space, the process remains there for all time, since $\mu _0=0$ and $\lambda _N=0$. The origin is seen to be an absorbing state, since both $\lambda _0$ and $\mu _0$ are equal to zero. Absorption at the origin corresponds to extinction of the population. To study a surviving population, we introduce the conditioned random variable $X^{(Q)}(t)$ by conditioning *X*(*t*) on the event $X(t)>0$. Thus,

5 $$\begin{aligned} P\{X^{(Q)}(t)=k\} = P\{X(t)=k|X(t)>0\}, \quad k=1,2,\dots ,N. \end{aligned}$$

The state space of the conditioned random variable $X^{(Q)}(t)$ differs from that of *X*(*t*) in one respect: The origin can be reached by *X*(*t*), but not by the conditioned random variable $X^{(Q)}(t)$. Thus, the state space of the latter one of these random variables is equal to $\{1,2,\dots ,N\}$. Stationary distributions of the two random variables are vastly different. The stationary distribution of *X*(*t*) is degenerate with probability one at the origin, while the stationary distribution of the conditioned random variable $X^{(Q)}(t)$ is the important quasi-stationary distribution (QSD), supported on the state space $\{1,2,\dots ,N\}$.

We shall use the second of the two model formulations in this paper. An important reason for this is that the second formulation contains a parameter *N* that can take large values. The presence of such a parameter is essential for the formulation of asymptotic approximations. Another advantage of the second formulation is that it gives information about dimensions of parameters and state variables. Such information is of value for biological interpretations of all quantities that appear in the study of the model.

The two formulations of the stochastic power law logistic model are essentially equivalent if we require in the first formulation that $(a_1/b_1)^{1/s}=N$ is an integer, that the state space is finite and equal to $\{0,1,2,\dots ,N\}$, and that $b_2 \ge 0$. We assume in what follows in this paper that these three requirements are met. The second model formulation can then be seen as a reparameterization of the first one. We find then that the four parameters $a_1, a_2, b_1, b_2$ of the first formulation can be expressed in terms of the five parameters $\mu , R_0, \alpha , N, s$ of the second formulation as follows:

6 $$\begin{aligned}&a_1 = \mu R_0, \end{aligned}$$

7 $$\begin{aligned}&a_2 = \mu , \end{aligned}$$

8 $$\begin{aligned}&b_1 = \mu \frac{R_0}{N^s}, \end{aligned}$$

9 $$\begin{aligned}&b_2 = \mu \frac{\alpha }{N^s}. \end{aligned}$$

Obviously, *s* takes the same value in both formulations. We note also that some results that we shall refer to below use the following notations for the sums and differences of the parameters $a_i$ and $b_i$:

10 $$\begin{aligned}&a = a_1 - a_2 = \mu (R_0-1), \end{aligned}$$

11 $$\begin{aligned}&b = b_1 + b_2 = \mu \frac{R_0+\alpha }{N^s}, \end{aligned}$$

12 $$\begin{aligned}&c = a_1 + a_2 = \mu (R_0+1), \end{aligned}$$

13 $$\begin{aligned}&d = b_1 - b_2 = \mu \frac{R_0-\alpha }{N^s}. \end{aligned}$$

As already mentioned, the logistic Verhulst model can be seen as a special case of the power law logistic model, corresponding to $s=1$. Asymptotic approximations of the first three cumulants of the QSD for the Verhulst model are derived in Nåsell (2017). The goal of the present paper is to derive corresponding approximations of the first three cumulants of the QSD of the power law logistic model for positive values of *s*. We proceed by first deriving asymptotic approximations of the first three cumulants of the QSD for the *s*-values 2, 3, and 4. These results are then extended in Sect. 7 to both integer and non-integer positive values of *s*.

## Numerical Evaluations

Numerical evaluations have been used in earlier work on this model, and we shall also use them here. However, there are differences between our approach and those of earlier workers both in the methods used for deriving numerical results, and in the type of evaluations that are carried out.

Bartlett et al. (1960) argue incorrectly that a stationary distribution cannot exist for the model that they are concerned with, since $\lambda _0=0$, while we claim that the process in this case has a degenerate stationary distribution with probability one at the origin. Bartlett et al. claim furthermore that “the probability distribution for the stationary (or quasi-stationary) distribution must satisfy the recurrence relation”

14 $$\begin{aligned} \mu _n p_n = \lambda _{n-1} p_{n-1}. \end{aligned}$$

We find it disturbing that they refer to both stationary and quasi-stationary distributions here, without clarifying which of these two distributions that they mean. The statement can certainly not hold for both of them. The fact is that it is incorrect for both of them. Instead, the relation (14) holds for the stationary distribution $p^{(0)}$ of a related auxiliary process $X^{(0)}(t)$ that is useful for studying the quasi-stationary distribution. The transition rates of this auxiliary process are equal to those of the process *X*(*t*), with the one exception that the rate $\mu _1$ for transition from the state 1 to the absorbing state 0 is replaced by zero. The stationary distribution $p^{(0)}$ is non-degenerate and can be evaluated explicitly. It has been used to study the QSD for a SIS model, which is the special case of the model that we are concerned with here that corresponds to the parameter values $s=1$ and $\alpha =0$, in Nåsell (2011). It follows from this study that $p^{(0)}$ is a good approximation of the QSD in its body, but not in the left tail, in the parameter region where $R_0>1$, but also that $p^{(0)}$ is not a good approximation of the QSD if $R_0<1$, nor in the parameter region near $R_0=1$. Similar relations between $p^{(0)}$ and the QSD are expected to hold for $\alpha >0$ and for integer values of *s* larger than 1. Both Bartlett et al. (1960) and Matis et al. (1998) use the recurrence relation (14) as a basis for numerical evaluations of the QSD. Our approach is different, as shown below. However, we expect the cumulants of the stationary distribution $p^{(0)}$ to be close to the cumulants of the QSD in the parameter region where $R_0>1$, since the tail probabilities have a minor influence on the cumulant values. We note that Renshaw (2011) uses $p^{(0)}$ as a “working definition” of quasi-stationarity. We do not follow this approach. Our results require a sharp line to be drawn between exact and approximate relations.

To describe our method for numerical evaluations, we need a second auxiliary process $X^{(1)}(t)$. The birth rates of this process are equal to those of the original process *X*(*t*), while the death rates are slightly smaller than those of *X*(*t*), and equal to $\mu _{n-1}$. This means that the second auxiliary process can be interpreted as the original process *X*(*t*) with one surviving and immortal individual. Like the first of the two auxiliary processes, the second one lacks absorbing state, and its stationary distribution $p^{(1)}$ is non-degenerate and can be evaluated explicitly.

Our numerical evaluations of the QSD make use of the restart map $\varPsi$ analyzed by Ferrari et al. (1995), and also discussed by Nåsell (2011). This restart map is defined as follows: Let a probability vector $\nu = (\nu _1, \nu _2, \dots , \nu _N)$ be given. Define a process related to the process *X*(*t*) that we are studying by the requirement that whenever the original process reaches the state zero, it is immediately restarted at some state *j* with probability $\nu _j$. The restarted process has the state space $\{1, 2, \dots , N\}$, and a unique stationary distribution *p*. The map $\varPsi$ is then defined by $\varPsi (\nu )=p$. The quasi-stationary distribution *q* is a fixed point of the mapping $\varPsi$, since $\varPsi (q)=q$. Furthermore, Ferrari et al. (1995) show that an iteration scheme can be established by repeated applications of the map $\varPsi$ to an arbitrary initial probability vector, and that it converges to the quasi-stationary distribution *q*. Suitable initial probability vectors are given by the stationary distributions $p^{(0)}$ and $p^{(1)}$ of the two auxiliary processes $X^{(0)}(t)$ and $X^{(1)}(t)$. It turns out that that the map $\varPsi$ can be described explicitly with the aid of these two stationary distributions. A derivation is given by Nåsell (2011). By denoting the components of the vector $p=\varPsi (\nu )$ by $p_n$, we find that

15 $$\begin{aligned} p_n = \pi _n S_n p_1, \quad n=1,2,\dots ,N, \end{aligned}$$

where

16 $$\begin{aligned} \pi _1=1, \quad \pi _n = \frac{\lambda _1\lambda _2\cdots \lambda _{n-1}}{\mu _2\mu _3\cdots \mu _n}, \quad n=2,3,\dots ,N, \end{aligned}$$

and

17 $$\begin{aligned} S_n = \sum _{k=1}^{n} \frac{1-\sum _{j=1}^{k-1}\nu _j}{\rho _k}, \quad n=1,2,\dots ,N, \end{aligned}$$

with

18 $$\begin{aligned} \rho _1=1, \quad \rho _n = \frac{\lambda _1\lambda _2\cdots \lambda _{n-1}}{\mu _1\mu _2\cdots \mu _{n-1}}, \quad n=2,3,\dots ,N, \end{aligned}$$

and

19 $$\begin{aligned} p_1 = \frac{1}{\sum _{n=1}^N \pi _n S_n}. \end{aligned}$$

The stationary distributions $p^{(0)}$ and $p^{(1)}$ of the two auxiliary processes are determined from the sequences $\pi _n$ and $\rho _n$ as follows:

20 $$\begin{aligned} p_n^{(0)} = \pi _n p_1^{(0)}, \quad n=1,2,\dots ,N, \quad \text {where} \quad p_1^{(0)} = \frac{1}{\sum _{n=1}^N \pi _n}, \end{aligned}$$

and

21 $$\begin{aligned} p_n^{(1)} = \rho _n p_1^{(1)}, \quad n=1,2,\dots ,N, \quad \text {where} \quad p_1^{(1)} = \frac{1}{\sum _{n=1}^N \rho _n}, \end{aligned}$$

Our numerical method for determining the quasi-stationary distribution consists in applying the restart map $\varPsi$ to a suitable initial distribution and continues iterations until successive iterates are sufficiently close. In case $R_0>1$, which is the parameter region of main interest for the study of cumulants in this paper, the stationary distribution $p^{(0)}$ is recommended over the distribution $p^{(1)}$ as initial distribution.

Numerical evaluations are used by Matis et al. (1998) for showing that the errors that their approximations lead to are small. With our different parameterizations, we have access to a parameter *N* that can take large values. We can then use numerical evaluations to extract additional information both about the model and about the approximations that we derive. One goal is to derive information about the orders of magnitude (in terms of *N*) of various cumulants, and another one concerns the probability of taking the value 1 in the quasi-stationary distribution. Information of these two types is important for the formulation of hypotheses as a basis for finding asymptotic approximations of the first few cumulants for large values of *N*, as will be shown below. Another use of numerical evaluations is to derive information about the orders of magnitude (in terms of *N*) of the errors that are committed in using our approximations. Such results will be used below to support the forms of asymptotic approximations that we derive.

## ODEs for Cumulants of the Unconditioned Random Variable *X*(*t*)

The starting point for our derivations of asymptotic approximations of the first three cumulants of the QSD is ODEs for the first three cumulants of the conditioned random variable $X^Q(t)$. It turns out that these ODEs are closely related to the ODEs for the corresponding cumulants of the unconditioned random variable *X*(*t*) in one important region of parameter space, namely where $R_0>1$. We start therefore by giving these latter ODEs.

The derivation of these ODEs makes use of the moment generating function $M(\theta ,t)$ and the cumulant generating function $K(\theta ,t)$ of the random variable *X*(*t*). They are defined by

22 $$\begin{aligned} M(\theta ,t) = E \exp (\theta X(t)) = \sum _{n=0}^N p_n(t) \exp (n\theta ) \end{aligned}$$

and

23 $$\begin{aligned} K(\theta ,t) = \log M(\theta ,t), \end{aligned}$$

where the state probabilities $p_n(t)=P\{X(t)=n\}$ satisfy the Kolmogorov forward equations

24 $$\begin{aligned} p_n'(t) = \lambda _{n-1} p_{n-1}(t) - (\lambda _n + \mu _n) p_n(t) + \mu _{n+1} p_{n+1}(t), \quad n = 0,1,\dots ,N.\nonumber \\ \end{aligned}$$

(Here, we agree to put $\lambda _{-1} = \mu _{N+1} = p_{-1}(t) = p_{N+1}(t) = 0$ so that (24) makes sense formally for all *n* values indicated.)

The definitions of the cumulant generating function *K* and of the transition rates $\lambda _ n$ and $\mu _n$ can be used to derive a partial differential equation (PDE) for *K*. It can be written as follows:

25 $$\begin{aligned} \frac{\partial K(\theta ,t)}{\partial t}= & {} \mu (\exp (\theta )-1) \left[ (R_0-\exp (-\theta )) \frac{\partial K(\theta ,t)}{\partial \theta } \right. \nonumber \\&\left. - \frac{R_0 + \alpha \exp (-\theta )}{N^s} \exp (-K(\theta ,t)) \frac{\partial ^{s+1}\exp (K(\theta ,t))}{\partial \theta ^{s+1}} \right] . \end{aligned}$$

The same result follows by applying the reparameterization in (6)–(9) to the expression for the PDE given by Renshaw (2011), and numbered (3.4.11).

The cumulants $\kappa _i(t)$ are obtained from a power series expansion of the cumulant generating function $K(\theta ,t)$ as follows:

26 $$\begin{aligned} K(\theta ,t) = \sum _{i=1}^{\infty } \frac{\kappa _i(t)}{i!} \theta ^i. \end{aligned}$$

We use this result for determining the ODEs of the first few cumulants of the random variable *X*(*t*). They are found by determining the first few terms of the expansion of the PDE of the cumulant generating function $K(\theta ,t)$ in terms of $\theta$ and then identifying terms with equal powers of $\theta$.

To express our results, we use the capital letters *A*, *B*, and *C* to denote the time derivatives of the first three cumulants of the random variable *X*(*t*), with superscripts indicating the *s*-values, as follows:

27 $$\begin{aligned} A^{(s)}(t)&= \kappa _1^{(s)^{'}}(t), \end{aligned}$$

28 $$\begin{aligned} B^{(s)}(t)&= \kappa _2^{(s)^{'}}(t), \end{aligned}$$

29 $$\begin{aligned} C^{(s)}(t)&= \kappa _3^{(s)^{'}}(t). \end{aligned}$$

Expressions for the functions $A^{(s)}, B^{(s)}, C^{(s)}$ for the *s*-values 2, 3 and 4 can then be written as follows:

30 $$\begin{aligned} A^{(2)}&= \mu (R_0-1) \kappa _1 - \mu \frac{R_0 + \alpha }{N^2} (\kappa _1^3 + 3 \kappa _1 \kappa _2 + \kappa _3), \end{aligned}$$

31 $$\begin{aligned} B^{(2)}&= 2 \mu (R_0-1) \kappa _2 + \mu (R_0+1)\kappa _1 - \mu \frac{R_0 - \alpha }{N^2} (\kappa _1^3 + 3 \kappa _1 \kappa _2 + \kappa _3) \nonumber \\&\quad - 2 \mu \frac{R_0 + \alpha }{N^2} (3 \kappa _1^2 \kappa _2 + 3 \kappa _1 \kappa _3 + 3 \kappa _2^2 + \kappa _4), \end{aligned}$$

32 $$\begin{aligned} C^{(2)}&= \mu (R_0-1) (\kappa _1 + 3 \kappa _3) + 3 \mu (R_0+1) \kappa _2\nonumber \\&\quad - 3 \mu \frac{R_0 - \alpha }{N^2} (3 \kappa _1^2 \kappa _2 + 3 \kappa _1 \kappa _3 + 3 \kappa _2^2 + \kappa _4) \nonumber \\&\quad - \mu \frac{R_0 + \alpha }{N^2} (\kappa _1^3 + 9 \kappa _1^2 \kappa _3 + 18 \kappa _1 \kappa _2^2 + 3 \kappa _1 \kappa _2 + 9 \kappa _1 \kappa _4 + 27 \kappa _2 \kappa _3 + \kappa _3 + 3 \kappa _5), \end{aligned}$$

33 $$\begin{aligned} A^{(3)}&= \mu (R_0-1) \kappa _1 - \mu \frac{R_0 + \alpha }{N^3} (\kappa _1^4 + 6 \kappa _1^2 \kappa _2 + 4 \kappa _1 \kappa _3 + 3 \kappa _2^2 + \kappa _4 ), \end{aligned}$$

34 $$\begin{aligned} B^{(3)}&= 2 \mu (R_0-1) \kappa _2 + \mu (R_0+1)\kappa _1 \nonumber \\&\quad - \mu \frac{R_0 - \alpha }{N^3} (\kappa _1^4 + 6 \kappa _1^2 \kappa _2 + 4 \kappa _1 \kappa _3 + 3 \kappa _2^2 + \kappa _4)\nonumber \\&\quad - 2 \mu \frac{R_0 + \alpha }{N^3} (4 \kappa _1^3 \kappa _2 + 6 \kappa _1^2 \kappa _3 + 12 \kappa _1 \kappa _2^2 + 4 \kappa _1 \kappa _4 + 10 \kappa _2 \kappa _3 + \kappa _5), \end{aligned}$$

35 $$\begin{aligned} C^{(3)}&= \mu (R_0-1) (\kappa _1 + 3 \kappa _3) + 3 \mu (R_0+1) \kappa _2\nonumber \\&\quad - 3 \mu \frac{R_0 - \alpha }{N^3} (4 \kappa _1^3 \kappa _2 + 6 \kappa _1^2 \kappa _3 + 12 \kappa _1 \kappa _2^2 + 4 \kappa _1 \kappa _4 + 10 \kappa _2 \kappa _3 + \kappa _5) \nonumber \\&\quad - \mu \frac{R_0 + \alpha }{N^3} (\kappa _1^4 + 12 \kappa _1^3 \kappa _3 + 36 \kappa _1^2 \kappa _2^2 + 6 \kappa _1^2 \kappa _2 + 18 \kappa _1^2 \kappa _4 + 108 \kappa _1 \kappa _2 \kappa _3\nonumber \\&\qquad + 4 \kappa _1 \kappa _3 + 12 \kappa _1 \kappa _5 + 36 \kappa _2^3 + 3 \kappa _2^2 + 42 \kappa _2 \kappa _4 + 30 \kappa _3^2 + \kappa _4 + 3 \kappa _6 ), \end{aligned}$$

36 $$\begin{aligned} A^{(4)}&= \mu (R_0-1) \kappa _1 \nonumber \\&\quad - \mu \frac{R_0+\alpha }{N^4}(\kappa _1^5 + 10 \kappa _1^3 \kappa _2 + 10 \kappa _1^2 \kappa _3 + 15 \kappa _1 \kappa _2^2 + 5 \kappa _1 \kappa _4 + 10 \kappa _2 \kappa _3 + \kappa _5), \end{aligned}$$

37 $$\begin{aligned} B^{(4)}&= 2 \mu (R_0-1)\kappa _2 + \mu (R_0+1) \kappa _1 \nonumber \\&\quad - \mu \frac{R_0-\alpha }{N^4}(\kappa _1^5 + 10\kappa _1^3 \kappa _2 + 10 \kappa _1^2 \kappa _3 + 15 \kappa _1 \kappa _2^2 + 5 \kappa _1 \kappa _4 + 10 \kappa _2 \kappa _3 + \kappa _5) \nonumber \\&\quad - 2 \mu \frac{R_0+\alpha }{N^4}(5\kappa _1^4 \kappa _2 + 10 \kappa _1^3 \kappa _3 + 30 \kappa _1^2 \kappa _2^2 + 10 \kappa _1^2 \kappa _4 + 50 \kappa _1 \kappa _2 \kappa _3 + 5 \kappa _1 \kappa _5 \nonumber \\&\quad + 15 \kappa _2^3 + 15 \kappa _2 \kappa _4 + 10 \kappa _3^2 + \kappa _6), \end{aligned}$$

38 $$\begin{aligned} C^{(4)}&= \mu (R_0-1)(\kappa _1 + 3 \kappa _3) + 3 \mu (R_0+1) \kappa _2 \nonumber \\&\quad - 3 \mu \frac{R_0-\alpha }{N^4} (5 \kappa _1^4 \kappa _2 + 10 \kappa _1^3 \kappa _3 + 30 \kappa _1^2 \kappa _2^2 + 10 \kappa _1^2 \kappa _4 + 50 \kappa _1 \kappa _2 \kappa _3 + 5 \kappa _1 \kappa _5\nonumber \\&\quad + 15 \kappa _2^3 + 15 \kappa _2 \kappa _4 + 10 \kappa _3^2 + \kappa _6) \nonumber \\&\quad - \mu \frac{R_0+ \alpha }{N^4} (\kappa _1^5 + 15 \kappa _1^4 \kappa _3 + 60 \kappa _1^3 \kappa _2^2 + 10 \kappa _1^3 \kappa _2 + 30 \kappa _1^3 \kappa _4 + 270 \kappa _1^2 \kappa _2 \kappa _3 \nonumber \\&\quad + 10 \kappa _1^2 \kappa _3 + 30 \kappa _1^2 \kappa _5 + 180 \kappa _1 \kappa _2^3 + 15 \kappa _1 \kappa _2^2 + 210 \kappa _1 \kappa _2 \kappa _4 \nonumber \\&\quad + 150 \kappa _1 \kappa _3^2 + 5 \kappa _1 \kappa _4 + 15 \kappa _1 \kappa _6 + 285 \kappa _2^2 \kappa _3 + 10 \kappa _2 \kappa _3 + 60 \kappa _2 \kappa _5 \nonumber \\&\quad + 105 \kappa _3 \kappa _4 + \kappa _5 + 3 \kappa _7). \end{aligned}$$

Derivations of these results using Maple are given in Nåsell (2018).

We note that the 3 derivatives $A^{(2)}, A^{(3)}, A^{(4)}$ in (30), (33), (36) of the first cumulant $\kappa _1$ for the 3 *s*-values 2, 3, and 4 are found from the expressions (27), (28), (29) in Matis et al. (1998) by reparameterization using (10)–(13) after introducing the correction that the first term $(a-b\kappa _1^4)$ in the right-hand side of (29) is written $(a-b\kappa _1^4)\kappa _1$. Similarly, the 2 derivatives $B^{(2)}$ and $B^{(3)}$ in (31) and (34) of the second cumulant $\kappa _2$ for the 2 *s*-values 2 and 3 are found from the expressions (33) and (34) in Matis et al. by the same reparameterization after noting that the minus sign of the term $-2a\kappa _2$ in the right-hand side of their formula (34) is incorrect and should be changed to a plus sign.

## ODEs for Cumulants of the Conditioned Random Variable $X^{(Q)}(t)$

The goal of our study is to determine approximations of the first three cumulants of the QSD for the stochastic variable *X*(*t*). This leads us to a study of the stationary values of the system of ODEs for the first three cumulants of the conditioned random variable $X^{(Q)}(t)$. These ODEs turn out to be closely related to the ODEs for the cumulants of the unconditioned random variable *X*(*t*) in one important parameter region, namely where $R_0>1$. Identification of this parameter region is of high importance, since it gives the validity condition for the approximations that we derive.

In similarity to the case in the previous section, we use the cumulant generating function $K^{(Q)}(\theta ,t)$ of the random variable $X^{(Q)}(t)$ that is of concern here to derive the ODEs of the first few cumulants of this random variable. To derive the PDE of this cumulant generating function, we proceed as in the previous section. The main difference from the case in the previous section is that the expression for the time derivative of the state probability $p_n^{(Q)}(t)$ is different from the counterpart of (24). By using the relation in (5), we find that

39 $$\begin{aligned} p_n^{(Q)}(t) = \frac{p_n(t)}{1-p_0(t)}, \quad n=1,2,\dots ,N. \end{aligned}$$

Differentiation and use of the Kolmogorov forward equations in (24) gives

40 $$\begin{aligned} {p_n^{(Q)}}'(t)= & {} \lambda _{n-1} p_{n-1}^{(Q)}(t) - (\lambda _n + \mu _n) p_n^{(Q)}(t) + \mu _{n+1} p_{n+1}^{(Q)}(t) \nonumber \\&+ \mu _1 p_1^{(Q)}(t) p_n^{(Q)}(t), \quad n=1,2,\dots ,N. \end{aligned}$$

The PDE for the cumulant generating function $K^{(Q)}(\theta ,t)$ of the conditioned random variable $X^Q(t)$ turns out to be quite similar to the PDE for $K(\theta ,t)$. It can be written as follows:

41 $$\begin{aligned} \frac{\partial K^{(Q)}(\theta ,t)}{\partial t}= & {} \mu (\exp (\theta )-1) \left[ (R_0-\exp (-\theta )) \frac{\partial K^{(Q)}(\theta ,t)}{\partial \theta } \right. \nonumber \\&\left. - \frac{R_0 + \alpha \exp (-\theta )}{N^s} \exp (-K^{(Q)}(\theta ,t)) \frac{\partial ^{s+1}\exp (K^{(Q)}(\theta ,t))}{\partial \theta ^{s+1}} \right] \nonumber \\&+ \mu \left( 1+\frac{\alpha }{N}\right) p_1^{(Q)}(t) \left( 1 - \exp (-K^{(Q)}(\theta ,t)\right) . \end{aligned}$$

As in the previous section, we determine the ODEs of the first few cumulants by expanding this PDE in terms of $\theta$ and identifying terms of equal powers of $\theta$. We find then that the last term of the right-hand side of every such ODE contains $p^{(Q)}_1(t)$ as a factor. The stationary value of this probability is equal to the quasi-stationary probability $q_1$. We claim that this probability is exponentially small in *N* for any $\alpha \ge 0$ and any positive value of *s*, integer or not, when $R_0>1$. Arguments that support this are given below. The last term can therefore be ignored when we are searching for asymptotic approximations of the critical points. An important consequence of this is that asymptotic approximations of the first few cumulants of the QSD for $R_0>1$ are found as stationary solutions of the ODEs in Sect. 4 for the corresponding cumulants of the unconditioned random variable *X*(*t*), with obvious rules for exclusion of spurious solutions. Derivations of these results are given below in Sect. 6.

We show first that $q_1$ is exponentially small in *N* when $R_0>1$, $\alpha \ge 0$, and $s=1$. It follows from Nåsell (2011) that this result is true for the SIS model, defined by $s=1$ and $\alpha =0$. Furthermore, it follows from Nåsell (2001a, b) that the same result holds also for the Verhulst model, with $s=1$ and $\alpha >0$. Support for the wider claim that $q_1$ is exponentially small in *N* for $R_0>1$, $\alpha \ge 0$, and any $s>0$ is given in Table 1, which shows numerically determined values of $q_1$ for $R_0=3$, $\alpha =1$, the *s*-values 0.5, 1, 2, 3, and the *N*-values 100, 200, and 400. The table shows that $q_1$ decreases strongly with both *s* and *N* for these values of $R_0$ and $\alpha$. It shows also that a doubling of the *N*-value for fixed values of $R_0$, $\alpha$, and *s* leads to an approximate squaring of the value of $q_1$, as would be expected when $q_1$ is exponentially small in *N*.

The condition that we have found under which $q_1$ is exponentially small in *N*, namely $R_0>1$, turns out to be the condition for the validity of the approximations of the first three cumulants of the QSD that we derive later. We note that a corresponding condition of validity has been missing from earlier results for the same model, based on cumulant closure.

**Table 1:** Numerical evaluations of the probability $q_1$ of the QSD of the stochastic power law logistic model

*s*	$N=100$	$N=200$	$N=400$
0.5	$1.04*10^{-4}$	$8.68*10^{-9}$	$3.60*10^{-17}$
1	$1.73*10^{-12}$	$8.04*10^{-25}$	$1.18*10^{-49}$
2	$7.55*10^{-23}$	$8.61*10^{-46}$	$7.88*10^{-92}$
3	$1.42*10^{-28}$	$2.36*10^{-57}$	$4.54*10^{-115}$

Results are shown for $R_0=3$, $\alpha =1$, the *s*-values 0.5, 1, 2, 3, and the *N*-values 100, 200, and 400. The results indicate that $q_1$ is exponentially small. Derivations of these results using Maple are given in Nåsell (2018)

## Cumulants of the QSD: First Results

This section is used to derive asymptotic approximations of the first three cumulants of the quasi-stationary distribution of the stochastic power law logistic model for the *s*-values 2, 3, and 4 and large *N*-values in the parameter region where $R_0>1$. The method that we use is similar to the one introduced in the study of the cumulants of the QSD of the Verhulst logistic model in Nåsell (2017). This latter study actually represents the special case with $s=1$ of the model that we study here. Our results are based on assumptions about the forms of asymptotic approximations of the first few cumulants for large values of *N*. For the Verhulst model with $s=1$ and $R_0>1$, we give arguments in Nåsell (2017) for assuming that the first four cumulants of the QSD are $\text {O(N)}$. The results make it easy to conjecture that all finite order cumulants of the QSD for $s=1$ are $\text {O}(N)$ in the parameter region $R_0>1$. It is tempting to guess that this holds also for integer values of *s* larger than 1.

**Table 2:** Numerical evaluations of the first seven cumulants of the QSD of the stochastic power law logistic model

*s*	Cumulant	$N=100$	$N=200$	$N=400$
1	$\kappa _1$	81.6	163	327
1	$\kappa _2$	16.7	33.3	66.3
1	$\kappa _3$	$-$ 13.8	$-$ 27.3	$-$ 54.3
1	$\kappa _4$	8.61	16.9	33.6
1	$\kappa _5$	$-$ 0.532	$-$ 0.620	$-$ 0.833
1	$\kappa _6$	$-$ 10.0	$-$ 21.0	$-$ 42.8
1	$\kappa _7$	17.8	38.3	79.0
4	$\kappa _1$	95.0	190	380
4	$\kappa _2$	4.93	9.74	19.3
4	$\kappa _3$	$-$ 4.80	$-$ 9.45	$-$ 18.8
4	$\kappa _4$	4.63	9.09	18.0
4	$\kappa _5$	$-$ 4.61	$-$ 8.98	$-$ 17.7
4	$\kappa _6$	5.52	10.5	20.5
4	$\kappa _7$	$-$ 10.2	$-$ 18.9	$-$ 36.6

Results are shown for $R_0=10$, $\alpha =1$, the *s*-values 1 and 4, and the *N*-values 100, 200, and 400. The results indicate that the first seven cumulants are all $\text {O}(N)$

In what follows in this section, we shall assume that the first $s+3$ cumulants of the QSD are $\text {O(N)}$ when $s=1, 2, 3, 4$, $\alpha \ge 0$, and $R_0>1$. As a basis for this, we refer to the numerical results in Table 2. It shows numerically determined values of the first seven cumulants of the QSD for the parameter values $R_0=10$ and $\alpha =1$, with the two *s*-values 1 and 4, and with the three *N*-values 100, 200, and 400. It is easy to verify from the table that all cumulants experience an approximate doubling both when *N* is doubled from 100 to 200 and also when *N* is doubled from 200 to 400. We take this as a strong indication that all of the cumulants considered are $\text {O}(N)$. The results in Table 2 are derived using Maple in Nåsell (2018), where also similar results are given for the *s*-values 2 and 3.

We study first the case $s=2$. The assumptions that the first five cumulants $\kappa _1-\kappa _5$ are $\text {O}(N)$ in the parameter region $R_0>1$ for $s =2$ are basic for our further assumptions that the first five cumulants have the following asymptotic behaviors for $R_0>1$ and $s=2$:

42 $$\begin{aligned}&\kappa _1 = x_1 N + x_2 + \frac{x_3}{N} + \text {O}\left( \frac{1}{N^2}\right) , \quad R_0>1, \quad s=2, \end{aligned}$$

43 $$\begin{aligned}&\kappa _2 = y_1 N + y_2 + \text {O}\left( \frac{1}{N}\right) , \quad R_0>1, \quad s=2, \end{aligned}$$

44 $$\begin{aligned}&\kappa _3 = z_1 N + \text {O}(1), \quad R_0>1, \quad s=2, \end{aligned}$$

45 $$\begin{aligned}&\kappa _4 = u_1 N + \text {O}(1), \quad R_0>1, \quad s=2, \end{aligned}$$

46 $$\begin{aligned}&\kappa _5 = u_2 N + \text {O}(1), \quad R_0>1, \quad s=2. \end{aligned}$$

The reason for including different numbers of terms in these asymptotic approximations will become apparent shortly. Thus, we have introduced eight unknowns, namely $x_1, x_2, x_3, y_1, y_2, z_1, u_1, u_2$. We proceed to determine the first six of them. By inserting the above asymptotic approximations of the first five cumulants into the expressions (30)–(32) for the functions $A^{(2)}, B^{(2)}, C^{(2)}$, we find that asymptotic approximations for them can be written as follows:

47 $$\begin{aligned}&A^{(2)} = A^{(2)}_1 N + A^{(2)}_2 + \frac{A^{(2)}_3}{N} + \text {O}\left( \frac{1}{N^2}\right) , \quad R_0>1, \quad s=2, \end{aligned}$$

48 $$\begin{aligned}&B^{(2)} = B^{(2)}_1 N + B^{(2)}_2 + \text {O}\left( \frac{1}{N}\right) , \quad R_0>1, \quad s=2, \end{aligned}$$

49 $$\begin{aligned}&C^{(2)} = C^{(2)}_1 N + \text {O}(1), \quad R_0>1, \quad s=2, \end{aligned}$$

where

50 $$\begin{aligned} A^{(2)}_1&= \mu (R_0-1) x_1 - \mu (R_0+\alpha ) x_1^3, \end{aligned}$$

51 $$\begin{aligned} A^{(2)}_2&=\mu (R_0-1) x_2 - 3\mu (R_0+\alpha ) (x_1^2 x_2 + x_1 y_1), \end{aligned}$$

52 $$\begin{aligned} A^{(2)}_3&= \mu (R_0-1) x_3 -\mu (R_0+\alpha ) (3 x_1^2 x_3 + 3 x_1 x_2^2 + 3 x_1 y_2 + 3 x_2 y_1 + z_1), \end{aligned}$$

53 $$\begin{aligned} B^{(2)}_1&= 2 \mu (R_0-1) y_1 +\mu (R_0+1) x_1 -\mu (R_0-\alpha ) x_1^3 - 6\mu (R_0 + \alpha ) x_1^2 y_1, \end{aligned}$$

54 $$\begin{aligned} B^{(2)}_2&= 2 \mu (R_0-1) y_2 + \mu (R_0+1) x_2 - 3\mu (R_0-\alpha ) (x_1^2 x_2 + x_1 y_1) \nonumber \\&\quad - 6 \mu (R_0+\alpha ) (x_1^2 y_2 + 2 x_1 x_2 y_1 + x_1 z_1 + y_1^2), \end{aligned}$$

55 $$\begin{aligned} C^{(2)}_1&= \mu (R_0-1) (x_1 + 3 z_1) + 3 \mu (R_0+1) y_1 - 9 \mu (R_0-\alpha ) x_1^2 y_1\nonumber \\&\quad - \mu (R_0+\alpha ) (x_1^3 + 9 x_1^2 z_1 + 18 x_1 y_1^2). \end{aligned}$$

The reason for including different numbers of terms in the assumed asymptotic approximations of the cumulants $\kappa _1 - \kappa _5$ can be understood with reference to the resulting asymptotic approximations of the quantities $A^{(2)}, B^{(2)}$, and $C^{(2)}$. We note for example from the expression (30) for $A^{(2)}$ that an additional term in the assumed asymptotic approximation of $\kappa _2$ would contribute a term of the order of $1/N^2$ to the asymptotic approximation of $A^{(2)}$. Inclusion of such an additional term would therefore be absorbed in the error term for the asymptotic approximation of $A^{(2)}$. We note from the expressions in (50)–(55) that only the first six of the eight coefficients introduced above appear in these expressions.

Our assumptions for $s=2$ that the first five cumulants have the asymptotic approximations given in (42)–(46) lead to the asymptotic approximations of $A^{(2)}$, $B^{(2)}$, $C^{(2)}$ in (47)–(49). These expressions are equal to the derivatives with respect to time of the first three cumulants. Setting them equal to zero gives conditions for the stationary points of the corresponding system of ODEs. Since we are working with approximations instead of exact results, we transform these conditions into the three requirements that $A^{(2)}=\text {O}(1/N^2)$, $B^{(2)}=\text {O}(1/N)$, $C^{(2)}=\text {O}(1)$. These requirements are satisfied by the basic conditions that the six expressions $A^{(2)}_1, A^{(2)}_2, A^{(2)}_3, B^{(2)}_1, B^{(2)}_2, C^{(2)}_1$ in (50)–(55) are equal to zero. We note that each of these expressions is a polynomial of degree three in the six unknown coefficients $x_1, x_2, x_3, y_1, y_2, z_1$. Our basic problem is to solve the six equations that are formed by setting the corresponding expressions equal to zero for the six unknown coefficients. Each equation is a polynomial of degree $s+1=3$ in the six unknown coefficients. Solution appears possible, since the number of equations equals the number of unknowns. Further inspection of the six equations reveals that considerable simplification can be achieved in the solution by determining the six unknowns in a definite order, as described below. The first advantage is that each of the equations to be solved contains only one unknown coefficient, and the second one is that all equations except the first one are linear in the coefficient to be solved for. The very first equation is of degree $s+1=3$ in the case we are considering here with $s=2$. All of the roots of the first equation except one are spurious solutions that turn out to be easy to identify and to delete. Whenever a solution is found for one of the coefficients, its value is immediately inserted into the remaining unsolved equations. The order of solution that leads to these pleasant results is as follows: First, the equation $A^{(2)}_1=0$ is solved for $x_1$. This equation has in this case, with $s=2$, $s+1=3$ solutions. Among them, we exclude $x_1=0$ and $x_1<0$ as the only spurious solutions that appear in this method. The solution found for $x_1$ is then inserted into the remaining five unsolved equations. After finding $x_1$, we proceed to solve the equation $B^{(2)}_1=0$ for $y_1$, the equation $A^{(2)}_2=0$ for $x_2$, the equation $C^{(2)}_1=0$ for $z_1$, the equation $B^{(2)}_2=0$ for $y_2$, and the equation $A^{(2)}_3=0$ for $x_3$. It is elementary to use these rules to solve for the six unknown coefficients. Alternative derivations using Maple are given in Nåsell (2018). The solutions are as follows:

56 $$\begin{aligned} x_1&= \left( \frac{R_0-1}{R_0+\alpha }\right) ^{1/2}, \quad R_0>1, \quad \alpha \ge 0, \quad s=2, \end{aligned}$$

57 $$\begin{aligned} x_2&= -\frac{3}{4} \frac{(\alpha +1)R_0}{(R_0+\alpha )(R_0-1)}, \quad R_0>1, \quad \alpha \ge 0, \quad s=2, \end{aligned}$$

58 $$\begin{aligned} x_3&= - \frac{(\alpha +1)R_0}{(R_0+\alpha )^2 (R_0-1)^2} \left( \frac{R_0+\alpha }{R_0-1} \right) ^{1/2} \nonumber \\&\quad \cdot \left[ \frac{7}{8} (R_0^2+\alpha )+\frac{13}{32}(\alpha +1)R_0\right] , \quad R_0>1, \quad \alpha \ge 0, \quad s=2, \end{aligned}$$

59 $$\begin{aligned} y_1&= \frac{1}{2} \frac{(\alpha +1)R_0}{(R_0+\alpha ) (R_0-1)} \left( \frac{R_0-1}{R_0+\alpha }\right) ^{1/2}, \quad R_0>1, \quad \alpha \ge 0, \quad s=2, \end{aligned}$$

60 $$\begin{aligned} y_2&= \frac{3}{4} \frac{(\alpha +1)R_0}{(R_0+\alpha )^2 (R_0-1)^2} (R_0^2+\alpha ), \quad R_0>1, \quad \alpha \ge 0, \quad s=2, \end{aligned}$$

61 $$\begin{aligned} z_1&= - \frac{(\alpha +1)R_0}{(R_0+\alpha )^2(R_0-1)^2} \left( \frac{R_0-1}{R_0+\alpha } \right) ^{1/2} \nonumber \\&\quad \cdot \left[ \frac{1}{2}(R_0^2+\alpha )-\frac{1}{4}(\alpha +1) R_0\right] , \quad R_0>1, \quad \alpha \ge 0, \quad s=2. \end{aligned}$$

The method used here for derivation of asymptotic approximations of the first three cumulants of the QSD for $s=2$ can in principle be followed for higher integer values of *s*. We proceed to consider the case when $s=3$. We begin by assuming that the first six cumulants have the following asymptotic behaviors for $R_0>1$ and $s=3$:

62 $$\begin{aligned}&\kappa _1 = x_1 N + x_2 + \frac{x_3}{N} + \text {O}\left( \frac{1}{N^2}\right) , \quad R_0>1, \quad s=3, \end{aligned}$$

63 $$\begin{aligned}&\kappa _2 = y_1 N + y_2 + \text {O}\left( \frac{1}{N}\right) , \quad R_0>1, \quad s=3, \end{aligned}$$

64 $$\begin{aligned}&\kappa _3 = z_1 N + \text {O}(1), \quad R_0>1, \quad s=3, \end{aligned}$$

65 $$\begin{aligned}&\kappa _4 = u_1 N + \text {O}(1), \quad R_0>1, \quad s=3, \end{aligned}$$

66 $$\begin{aligned}&\kappa _5 = u_2 N + \text {O}(1), \quad R_0>1, \quad s=3, \end{aligned}$$

67 $$\begin{aligned}&\kappa _6 = u_3 N + \text {O}(1), \quad R_0>1, \quad s=3. \end{aligned}$$

Insertions of these asymptotic approximations of the first six cumulants into the expressions (33)–(35) for the functions $A^{(3)}, B^{(3)}, C^{(3)}$ lead to the following asymptotic approximations for them:

68 $$\begin{aligned}&A^{(3)} = A^{(3)}_1 N + A^{(3)}_2 + \frac{A^{(3)}_3}{N} + \text {O}\left( \frac{1}{N^2}\right) , \quad R_0>1, \quad s=3, \end{aligned}$$

69 $$\begin{aligned}&B^{(3)} = B^{(3)}_1 N + B^{(3)}_2 + \text {O}\left( \frac{1}{N}\right) , \quad R_0>1, \quad s=3, \end{aligned}$$

70 $$\begin{aligned}&C^{(3)} = C^{(3)}_1 N + \text {O}(1), \quad R_0>1, \quad s=3, \end{aligned}$$

where

71 $$\begin{aligned} A^{(3)}_1&= \mu (R_0-1) x_1 - \mu (R_0+\alpha ) x_1^4, \end{aligned}$$

72 $$\begin{aligned} A^{(3)}_2&= \mu (R_0-1) x_2 - 2\mu (R_0+\alpha ) (2x_1^3 x_2 +3x_1^2 y_1), \end{aligned}$$

73 $$\begin{aligned} A^{(3)}_3&= \mu (R_0-1) x_3 -\mu (R_0+\alpha ) (4 x_1^3 x_3 + 6 x_1^2 x_2^2 + 6 x_1^2 y_2 + 12 x_1 x_2 y_1 \nonumber \\&\quad + 4 x_1 z_1+ 3 y_1^2), \end{aligned}$$

74 $$\begin{aligned} B^{(3)}_1&= 2 \mu (R_0-1) y_1 + \mu (R_0+1) x_1 - \mu (R_0-\alpha ) x_1^4 - 8 \mu (R_0 + \alpha ) x_1^3 y_1, \end{aligned}$$

75 $$\begin{aligned} B^{(3)}_2&= 2 \mu (R_0-1) y_2 + \mu (R_0+1) x_2 - \mu (R_0-\alpha ) (4 x_1^3 x_2 + 6 x_1^2 y_1) \nonumber \\&\quad - \mu (R_0+\alpha ) (8 x_1^3 y_2 + 24 x_1^2 x_2 y_1 + 12 x_1^2 z_1 + 24 x_1 y_1^2), \end{aligned}$$

76 $$\begin{aligned} C^{(3)}_1&= \mu (R_0-1) (x_1 + 3 z_1) + 3 \mu (R_0+1) y_1 - 12 \mu (R_0-\alpha ) x_1^3 y_1 \nonumber \\&\quad - \mu (R_0+\alpha ) (x_1^4 + 12 x_1^3 z_1 + 36 x_1^2 y_1^2). \end{aligned}$$

These six expressions are all polynomials of degree four in the six unknowns $x_1, x_2, x_3, y_1, y_2, z_1$. We solve the six equations formed by putting each of these expressions equal to zero for the six unknowns $x_1, x_2, x_3, y_1, y_2, z_1$. First, the equation $A^{(3)}_1=0$ is solved for $x_1$. Among the four roots, we exclude the one that is equal to zero, and the two that are complex conjugate as the only spurious solutions that appear in this method. The remaining five equations are then solved for the remaining five unknowns, using an order of solution similar to what is described for the case $s=2$ above. Derivations using Maple are given in Nåsell (2018). The results are as follows:

77 $$\begin{aligned} x_1&= \left( \frac{R_0-1}{R_0+\alpha }\right) ^{1/3}, \quad R_0>1, \quad \alpha \ge 0, \quad s=3, \end{aligned}$$

78 $$\begin{aligned} x_2&= -\frac{2}{3} \frac{(\alpha +1)R_0}{(R_0+\alpha )(R_0-1)}, \quad R_0>1, \quad \alpha \ge 0, \quad s=3, \end{aligned}$$

79 $$\begin{aligned} x_3&= - \frac{(\alpha +1)R_0}{(R_0+\alpha )^2(R_0-1)^2} \left( \frac{R_0+\alpha }{R_0-1} \right) ^{1/3} \nonumber \\&\quad \cdot \left[ \frac{8}{9}(R_0^2+\alpha ) +\frac{7}{27}(\alpha +1)R_0\right] , \quad R_0>1, \quad \alpha \ge 0, \quad s=3 \end{aligned}$$

80 $$\begin{aligned} y_1&= \frac{1}{3} \frac{(\alpha +1)R_0}{(R_0+\alpha ) (R_0-1)} \left( \frac{R_0-1}{R_0+\alpha } \right) ^{1/3}, \quad R_0>1, \quad \alpha \ge 0, \quad s=3, \end{aligned}$$

81 $$\begin{aligned} y_2&= \frac{2}{3} \frac{(\alpha +1)R_0}{(R_0+\alpha )^2 (R_0-1)^2}(R_0^2+\alpha ), \quad R_0>1, \quad \alpha \ge 0, \quad s=3, \end{aligned}$$

82 $$\begin{aligned} z_1&= - \frac{(\alpha +1)R_0}{(R_0+\alpha )^2(R_0-1)^2} \left( \frac{R_0-1}{R_0+\alpha }\right) ^{1/3} \nonumber \\&\quad \cdot \left[ \frac{1}{3}(R_0^2+\alpha ) -\frac{1}{9}(\alpha +1)R_0\right] , \quad R_0>1, \quad \alpha \ge 0, \quad s=3. \end{aligned}$$

The last problem addressed in this section is the derivation of asymptotic approximations of the first three cumulants of the QSD for $s=4$. We begin by assuming that the first seven cumulants have the following asymptotic behaviors for $R_0>1$ and $s=4$:

83 $$\begin{aligned}&\kappa _1 = x_1 N + x_2 + \frac{x_3}{N} + \text {O}\left( \frac{1}{N^2}\right) , \quad R_0>1, \quad s=4, \end{aligned}$$

84 $$\begin{aligned}&\kappa _2 = y_1 N + y_2 + \text {O}\left( \frac{1}{N}\right) , \quad R_0>1, \quad s=4, \end{aligned}$$

85 $$\begin{aligned}&\kappa _3 = z_1 N + \text {O}(1), \quad R_0>1, \quad s=4, \end{aligned}$$

86 $$\begin{aligned}&\kappa _4 = u_1 N + \text {O}(1), \quad R_0>1, \quad s=4, \end{aligned}$$

87 $$\begin{aligned}&\kappa _5 = u_2 N + \text {O}(1), \quad R_0>1, \quad s=4, \end{aligned}$$

88 $$\begin{aligned}&\kappa _6 = u_3 N + \text {O}(1), \quad R_0>1, \quad s=4, \end{aligned}$$

89 $$\begin{aligned}&\kappa _7 = u_4 N + \text {O}(1), \quad R_0>1, \quad s=4. \end{aligned}$$

Insertions of these asymptotic approximations of the first seven cumulants into the expressions (36)–(38) for the functions $A^{(4)}, B^{(4)}, C^{(4)}$ lead to the following asymptotic approximations for these three functions:

90 $$\begin{aligned}&A^{(4)} = A^{(4)}_1 N + A^{(4)}_2 + \frac{A^{(4)}_3}{N} + \text {O}\left( \frac{1}{N^2}\right) , \quad R_0>1, \quad s=4, \end{aligned}$$

91 $$\begin{aligned}&B^{(4)} = B^{(4)}_1 N + B^{(4)}_2 + \text {O}\left( \frac{1}{N}\right) , \quad R_0>1, \quad s=4, \end{aligned}$$

92 $$\begin{aligned}&C^{(4)} = C^{(4)}_1 N + \text {O}(1), \quad R_0>1, \quad s=4, \end{aligned}$$

where

93 $$\begin{aligned} A^{(4)}_1&= \mu (R_0-1) x_1 - \mu (R_0+\alpha ) x_1^5, \end{aligned}$$

94 $$\begin{aligned} A^{(4)}_2&= \mu (R_0-1) x_2 - 5 \mu (R_0+\alpha ) (x_1^4 x_2 + 2 x_1^3 y_1), \end{aligned}$$

95 $$\begin{aligned} A^{(4)}_3&= \mu (R_0-1) x_3 - 5 \mu (R_0+\alpha ) (x_1^4 x_3 + 2 x_1^3 x_2^2 + 2 x_1^3 y_2 + 6 x_1^2 x_2 y_1 \nonumber \\&\quad + 2 x_1^2 z_1 + 3 x_1 y_1^2), \end{aligned}$$

96 $$\begin{aligned} B^{(4)}_1&= 2\mu (R_0-1)y_1 + \mu (R_0+1) x_1 - \mu (R_0-\alpha ) x_1^5 -10 \mu (R_0+\alpha ) x_1^4 y_1, \end{aligned}$$

97 $$\begin{aligned} B^{(4)}_2&= 2 \mu (R_0-1) y_2 + \mu (R_0+1) x_2 - 5 \mu (R_0-\alpha ) (x_1^4 x_2 + 2 x_1^3 y_1) \nonumber \\&\quad - 10 \mu (R_0+\alpha ) (x_1^4 y_2 + 4 x_1^3 x_2 y_1 + 2 x_1^3 z_1 + 6 x_1^2 y_1^2), \end{aligned}$$

98 $$\begin{aligned} C^{(4)}_1&= \mu (R_0-1) (x_1 + 3 z_1) + 3 \mu (R_0+1) y_1 - 15 \mu (R_0-\alpha ) x_1^4 y_1 \nonumber \\&\quad - \mu (R_0+\alpha ) (x_1^5 + 15 x_1^4 z_1 + 60 x_1^3 y_1^2). \end{aligned}$$

These six expressions are all polynomials of degree five in the six unknown quantities $x_1, x_2, x_3, y_1, y_2, z_1$. As above, we solve the six equations formed by putting each of these expressions equal to zero for the six unknowns $x_1, x_2, x_3, y_1, y_2, z_1$. First, the equation $A^{(4)}_1 = 0$ is solved for $x_1$. Among the five roots, we exclude four of them as spurious solutions, namely one that is equal to zero, one that is negative, and two that are imaginary. The remaining five equations are then solved for the remaining five unknowns, using the same order as above for the cases $s=2$ and $s=3$. Derivations using Maple are given by Nåsell (2018). The results are as follows:

99 $$\begin{aligned} x_1&= \left( \frac{R_0-1}{R_0+\alpha }\right) ^{1/4}, \quad R_0>1, \quad \alpha \ge 0, \quad s=4, \end{aligned}$$

100 $$\begin{aligned} x_2&= - \frac{5}{8} \frac{(\alpha +1)R_0}{(R_0+\alpha )(R_0-1)}, \quad R_0>1, \quad \alpha \ge 0, \quad s=4, \end{aligned}$$

101 $$\begin{aligned} x_3&= - \frac{(\alpha +1)R_0}{(R_0+\alpha )^2 (R_0-1)^2} \left( \frac{R_0+\alpha }{R_0-1} \right) ^{1/4} \nonumber \\&\quad \cdot \left[ \frac{15}{16}(R_0^2+\alpha ) + \frac{25}{128}(\alpha +1)R_0 \right] , \quad R_0>1, \quad \alpha \ge 0,\quad s=4, \end{aligned}$$

102 $$\begin{aligned} y_1&= \frac{1}{4} \frac{(\alpha +1)R_0}{(R_0+\alpha ) (R_0-1)} \left( \frac{R_0-1}{R_0+\alpha }\right) ^{1/4}, \quad R_0>1, \quad \alpha \ge 0,\quad s=4, \end{aligned}$$

103 $$\begin{aligned} y_2&= \frac{5}{8} \frac{(\alpha +1)R_0}{(R_0+\alpha )^2(R_0-1)^2} (R_0^2+\alpha ), \quad R_0>1, \quad \alpha \ge 0, \quad s=4, \end{aligned}$$

104 $$\begin{aligned} z_1&= - \frac{(\alpha +1)R_0}{(R_0+\alpha )^2(R_0-1)^2} \left( \frac{R_0-1}{R_0+\alpha } \right) ^{1/4} \nonumber \\&\quad \cdot \left[ \frac{1}{4} (R_0^2+\alpha ) - \frac{1}{16}(\alpha +1)R_0 \right] , \quad R_0>1, \quad \alpha \ge 0, \quad s=4. \end{aligned}$$

## Cumulants of the QSD: Extensions to Positive Values of *s*

The results derived in the previous section give asymptotic approximations of the first three cumulants of the QSD for the integer *s*-values 2, 3, and 4. The present section is used to extend these approximations to positive values of *s*, both integer and non-integer. The extension to positive integer values of *s* starts out by giving asymptotic approximations of the first three cumulants of the QSD for the integer *s*-values from 1 to 10. For this, we use the method spelled out in detail in the previous section. It turns out that the *s*-dependence of these results can be described with the aid of five functions of *s* that all can be expressed as ratios of polynomials in *s* of degree two. After these five functions have been determined, we indicate that they can be used to extend our results to arbitrary positive integer values of *s*. No further evaluations are necessary for the extension of these results to positive non-integer values of *s*. We argue that small changes in the parameter *s* will lead to small changes in the first few cumulants of the QSD. The corresponding continuity argument lies behind our claim that the results that have been derived for positive integer values of *s* are valid for all positive values of *s*.

For easy reference we quote first from Nåsell (2017), the expressions for the six coefficients $x_1, x_2, x_3, y_1, y_2, z_1$ in the case $s=1$:

105 $$\begin{aligned}&x_1 = \frac{R_0-1}{R_0+\alpha }, \quad R_0>1, \quad \alpha \ge 0, \quad s=1, \end{aligned}$$

106 $$\begin{aligned}&x_2 = - \frac{(\alpha +1)R_0}{(R_0+\alpha )(R_0-1)}, \quad R_0>1, \quad \alpha \ge 0, \quad s=1, \end{aligned}$$

107 $$\begin{aligned}&x_3 = - \frac{(\alpha +1)R_0}{(R_0-1)^3} (R_0+1), \quad R_0>1, \quad \alpha \ge 0, \quad s=1, \end{aligned}$$

108 $$\begin{aligned}&y_1 = \frac{(\alpha +1)R_0}{(R_0+\alpha )^2}, \quad R_0>1, \quad \alpha \ge 0, \quad s=1, \end{aligned}$$

109 $$\begin{aligned}&y_2 = \frac{(\alpha +1)R_0}{(R_0+\alpha )^2 (R_0-1)^2} (R_0^2+\alpha ), \quad R_0>1, \quad \alpha \ge 0, \quad s=1, \end{aligned}$$

110 $$\begin{aligned}&z_1 = - \frac{(\alpha +1)R_0}{(R_0+\alpha )^3} (R_0-\alpha ) , \quad R_0>1, \quad \alpha \ge 0, \quad s=1. \end{aligned}$$

The asymptotic approximations of the first three cumulants of the QSD for the stochastic power law logistic model are given in terms of the following expressions for the coefficients $x_1, x_2, x_3, y_1, y_2, z_1$ for any $s>0$:

111 $$\begin{aligned} x_1&= \left( \frac{R_0-1}{R_0+\alpha } \right) ^{h_1(s)}, \quad R_0>1, \quad \alpha \ge 0, \quad s > 0, \end{aligned}$$

112 $$\begin{aligned} x_2&= - h_2(s) \frac{(\alpha +1) R_0}{(R_0+\alpha )(R_0-1)}, \quad R_0>1, \quad \alpha \ge 0, \quad s > 0, \end{aligned}$$

113 $$\begin{aligned} x_3&= - \frac{(\alpha +1)R_0}{(R_0+\alpha )^2(R_0-1)^2} \left( \frac{R_0+\alpha }{R_0-1} \right) ^{h_1(s)} \nonumber \\&\quad \cdot \left[ h_3(s) (R_0^2+\alpha )+h_4(s)(\alpha +1)R_0\right] , \quad R_0>1, \quad \alpha \ge 0, \quad s > 0, \end{aligned}$$

114 $$\begin{aligned} y_1&= h_1(s) \frac{(\alpha +1)R_0}{(R_0+\alpha )(R_0-1)} \left( \frac{R_0-1}{R_0+\alpha } \right) ^{h_1(s)}, \quad R_0>1, \quad \alpha \ge 0, \quad s > 0, \end{aligned}$$

115 $$\begin{aligned} y_2&= h_2(s) \frac{(\alpha +1)R_0}{(R_0+\alpha )^2(R_0-1)^2} (R_0^2+\alpha ), \quad R_0>1, \quad \alpha \ge 0, \quad s > 0, \end{aligned}$$

116 $$\begin{aligned} z_1&= -\frac{(\alpha +1)R_0}{(R_0+\alpha )^2(R_0-1)^2} \left( \frac{R_0-1}{R_0+\alpha } \right) ^{h_1(s)} \nonumber \\&\quad \cdot \left[ h_1(s) (R_0^2+\alpha ) - h_5(s)(\alpha +1)R_0 \right] , \quad R_0>1, \quad \alpha \ge 0, \quad s > 0, \end{aligned}$$

where the functions $h_1 - h_5$ are defined as follows:

117 $$\begin{aligned} h_1(s)&= \frac{1}{s}, \end{aligned}$$

118 $$\begin{aligned} h_2(s)&= \frac{s+1}{2s}, \end{aligned}$$

119 $$\begin{aligned} h_3(s)&= \frac{s^2 + 6s + 5}{12 s}, \end{aligned}$$

120 $$\begin{aligned} h_4(s)&= \frac{s^2 + 12s +11}{24 s^2}, \end{aligned}$$

121 $$\begin{aligned} h_5(s)&= \frac{1}{s^2}. \end{aligned}$$

To show that the functions $h_1-h_5$ are determined by these expressions for $s > 0$, we determine first the values that they take for the integer *s*-values 1–10, and given in Table 3. The values taken by these five functions for the integer *s*-values 1-4 are readily found from the expressions found for the 6 coefficients $x_1, x_2, x_3, y_1, y_2, z_1$ given in the previous and present sections. The further results for the *s*-values from 5 to 10 are based on asymptotic approximations of the first three cumulants of the QSD of the power law logistic model that have been derived using Maple, and are reported in Nåsell (2018).

It is straightforward to use the entries in Table 3 for the functions $h_1$, $h_2$, and $h_5$ to confirm that the expressions (117), (118), (121) for these functions are valid for all integer values of *s* from 1 to 10.

**Table 3:** Values of the functions $h_1 - h_5$ when their argument *s* takes integer values from 1 to 10. Derivations for *s* from 5 to 10 are given in Nåsell (2018)

*s*	$h_1(s)$	$h_2(s)$	$h_3(s)$	$h_4(s)$	$h_5(s)$
1	1	1	1	1	1
2	1/2	3/4	7/8	13/32	1/4
3	1/3	2/3	8/9	7/27	1/9
4	1/4	5/8	15/16	25/128	1/16
5	1/5	3/5	1	4/25	1/25
6	1/6	7/12	77/72	119/864	1/36
7	1/7	4/7	8/7	6/49	1/49
8	1/8	9/16	39/32	57/512	1/64
9	1/9	5/9	35/27	25/243	1/81
10	1/10	11/20	11/8	77/800	1/100

We turn now to deal with the functions $h_3$ and $h_4$. To find expressions for them, we assume that each of them can be written as a quotient of two polynomials in *s* of degree 2. To be specific, we assume that

122 $$\begin{aligned} h_3(s) = \frac{a_0 + a_1 s + a_2 s^2}{b_0 + b_1 s + b_2 s^2}, \end{aligned}$$

123 $$\begin{aligned} h_4(s) = \frac{c_0 + c_1 s + c_2 s^2}{d_0 + d_1 s + d_2 s^2}. \end{aligned}$$

To determine the values of the six coefficients $a_0, a_1, a_2, b_0, b_1, b_2$, we establish six equations that are found by equating the values of $h_3(s)$ for the integer *s*-values from 1 to 6 according to (122) to the corresponding values in Table 3. By solving these equations using Maple, as seen in Nåsell (2018), we find that $h_3(s)$ equals the expression in (119). An entirely similar treatment of the function $h_4$ leads to the expression (120) for $h_4(s)$. So far, we can conclude that the expressions (119) and (120) for $h_3(s)$ and $h_4(s)$ are valid for the integer *s*-values in the interval from 1 to 6. Extensions of the validities of the two expressions for $h_3(s)$ and $h_4(s)$ to all integer values of *s* from 1 to 10 are easily seen to hold by the simple expedient of verifying equalities between the values of the expressions in (119) and (120) and the corresponding values in Table 3. We take this as a strong indication that the domain of validity of the expressions (119) and (120) can be further extended to all positive integer values of *s*.

As a last step of extension, we use a continuity argument to claim that the domain of validity for the five functions $h_1 - h_5$ can be extended from all positive integers *s* to all positive values of *s*. We illustrate the result for non-integer *s*-values by giving the numerical values of the error terms for the three cumulants $\kappa _1, \kappa _2, \kappa _3$ in case $R_0=10$ and $\alpha =1$, for the two *s*-values 0.5 and 3.5, and the 3 *N*-values 100, 200, and 400 in Table 4. The table indicates that the error term for $\kappa _1$ is divided by approximately 4 for each doubling of *N*, the error term for $\kappa _2$ is divided by approximately 2 for each doubling of *N*, and the error term for $\kappa _3$ is approximately constant when *N* is doubled. This is consistent with the claims that the error terms of $\kappa _k$ are $\text {O}(1/N^{3-k})$ for the *k*-values 1, 2, and 3. Evaluations of the error terms in Table 4 are derived using Maple in Nåsell (2018).

**Table 4:** Numerical evaluations of the error terms of the approximations of the first three cumulants of the QSD of the stochastic power law logistic model

*s*	Cumulant	$$N=100$$	$$N=200 $$	$$N=400 $$
0.5	$\kappa _1$	$-227*10^{-6}$	$-55*10^{-6}$	$-14*10^{-6}$
0.5	$\kappa _2$	$111*10^{-4}$	$54*10^{-4}$	$27*10^{-4}$
0.5	$\kappa _3$	$-35*10^{-2}$	$-34*10^{-2}$	$-33*10^{-2}$
3.5	$\kappa _1$	$-334*10^{-7}$	$-83*10^{-7}$	$-21*10^{-7}$
3.5	$\kappa _2$	$247*10^{-5}$	$122*10^{-5}$	$61*10^{-5}$
3.5	$\kappa _3$	$-144*10^{-3}$	$-142*10^{-3}$	$-141*10^{-3}$

Results are shown for $R_0=10$, $\alpha =1$, the *s*-values 0.5 and 3.5, and the *N*-values 100, 200, and 400. The table indicates that the error term of $\kappa _1$ is $\text {O}(1/N^2)$, the error term of $\kappa _2$ is $\text {O}(1/N)$, and the error term of $\kappa _3$ is $\text {O}(1)$

The results in (111)–(116) show the way in which our asymptotic approximations of the first three cumulants of the QSD of the stochastic power law logistic model depend on the four parameters *N*, $R_0$, $\alpha$, and *s*. The one-term asymptotic approximations of the first three cumulants $\kappa _1$, $\kappa _2$, and $\kappa _3$ are useful for the information that they give about the behaviors of these cumulants. They can be written as follows:

124 $$\begin{aligned} \kappa _1&= x_1 N + O(1), \quad R_0>1, \quad \alpha \ge 0, \quad s > 0, \quad N\rightarrow \infty , \end{aligned}$$

125 $$\begin{aligned} \kappa _2&= y_1 N + O(1), \quad R_0>1, \quad \alpha \ge 0, \quad s > 0, \quad N\rightarrow \infty , \end{aligned}$$

126 $$\begin{aligned} \kappa _3&= z_1 N + O(1), \quad R_0>1, \quad \alpha \ge 0, \quad s > 0, \quad N\rightarrow \infty , \end{aligned}$$

where expressions for the three coefficients $x_1, y_1, z_1$ are given in (111), (114), and (116), respectively. We use these expressions to derive some properties of the first three cumulants, valid for sufficiently large values of *N*.

We note first that the expectation $\kappa _1$ is an increasing function of the power *s*. This follows from the following expression for the derivative of $x_1$ with respect to *s*:

127 $$\begin{aligned} \frac{\mathrm{d}x_1}{\mathrm{d}s} = \frac{1}{s^2} \log \left( \frac{R_0+\alpha }{R_0-1} \right) \left( \frac{R_0-1}{R_0+\alpha } \right) ^{1/s}. \end{aligned}$$

Our second observation concerns the variance $\kappa _2$. For sufficiently large values of *N*, we find that it has a maximum as a function of *s* at $s=s_2$, where

128 $$\begin{aligned} s_2 = \log ((R_0+\alpha )/(R_0-1)). \end{aligned}$$

This follows from the following expression for the derivative of $y_1$ with respect to *s*:

129 $$\begin{aligned} \frac{\mathrm{d}y_1}{\mathrm{d}s} = - \frac{1}{s^3} \frac{(\alpha +1)R_0}{(R_0+\alpha )(R_0-1)} \left( \frac{R_0-1}{R_0+\alpha } \right) ^{1/s} \left( s - \log \frac{R_0+\alpha }{R_0-1} \right) . \end{aligned}$$

We conclude in particular that $\kappa _2$ is increasing in *s* for $s < s_2$ and decreasing in *s* for $s>s_2$, provided *N* is large enough.

**Fig. 1:**
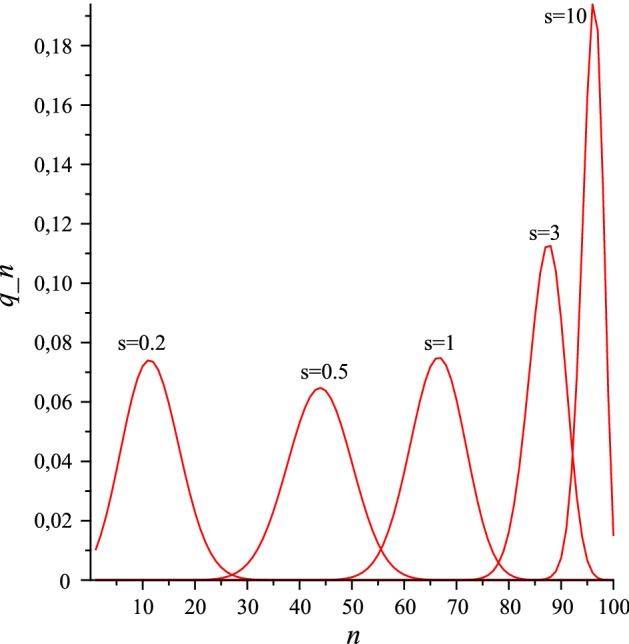
The QSD for the stochastic power law logistic model with $N=100$, $R_0=5$, $\alpha =1$, and the 5 *s*-values 0.2, 0.5, 1, 3, and 10

Our third observation concerns the third cumulant $\kappa _3$. It is useful for determining the skewness of the QSD. Actually, the QSD has negative skewness if $\kappa _3$ is negative, and positive skewness if $\kappa _3$ is positive. It follows from the expression (116) that $z_1$ is positive if $s<s_3$ and negative if $s>s_3$, where

130 $$\begin{aligned} s_3=(\alpha +1)R_0/(R_0^2+\alpha ). \end{aligned}$$

It is easy too see that the same intervals of *s* lead to positive, respectively negative, skewness of the QSD if *N* is sufficiently large.

Our brief study of the *s*-dependence of the first three cumulants shows that one can identify two cases with rather different behaviors. The first case occurs when $s<\min (s_2,s_3)$, and the second one when $s>\max (s_2,s_3)$. In the first case, we conclude that both $\kappa _1$ and $\kappa _2$ are increasing functions of *s*, and that the QSD has positive skewness. In the second case, we conclude as in the first case that $\kappa _1$ is increasing in *s*, while $\kappa _2$ now is a decreasing function of *s* and the skewness of the QSD has changed from positive to negative.

We illustrate the *s*-dependence of the first three cumulants by plotting the QSDs for five different *s*-values with the constant parameter values $N=100$, $R_0=5$, and $\alpha =1$. In these cases, we find $s_2=0.4055$ and $s_3=0.3846$. Numerically determined QSDs are shown in Fig. 1 for the five *s*-values 0.2, 0.5, 1, 3, and 10. The figure shows that the first cumulant (the expectation) increases as a function of *s*, that the second cumulant (the variance) increases as a function of *s* for small *s*, but decreases as a function of *s* for large *s*, and finally that the third cumulant (a measure of skewness) is positive for small *s*-values, and negative for large *s*-values. (For the interpretations of the plots in Fig. 1, it is useful to note that the probabilities $q_n$ are positive for all *n* in the state space $\{1,2,\dots ,N\}$. Recall that the QSDs are discrete, and that the individual probabilities $q_n$ are determined from the plotted curves by reading off the values at each abscissa *n*.)

The asymptotic approximations that we have given of the first three cumulants are valid for sufficiently large *N*-values. It is seen from the plot in Fig. 1 that the requirement that $q_1$ is exponentially small is not satisfied for $s=0.2$ when $R_0=5$, $N=100$ and $\alpha =1$. Clearly, a larger value of *N* is needed here for $q_1$ to be exponentially small in *N*.

## Discussion of Other Approaches

In this section, we discuss five published results that all deal with the problem in this paper, namely to determine approximations of the first three cumulants of the QSD of the stochastic power law logistic model. All these results are formally different from each other and also different from the results that we have derived and presented in Sect. 7 of this paper. We use the powerful method of asymptotic approximations to analyze these results. In this discussion, we are mainly limited to the case $s=1$.

The first published results are those due to Bartlett (1957) and Bartlett et al. (1960). The latter results are referred to as the BGL approximations. The model they analyze is the first one formulated in Sect. 2. Their derivation is based on a study of the changes of the stochastic variable *X*(*t*), and of its second and third powers, during an infinitesimal time interval. This requires approximations for which validity and error magnitudes have not been analyzed. The resulting approximations of the first three cumulants of the QSD are denoted by $\kappa _1^{(B)}$, $\kappa _2^{(B)}$, and $\kappa _3^{(B)}$, respectively. They are given as follows in Bartlett et al. (1960):

131 $$\begin{aligned} \kappa _1^{(B)}&= \frac{a_1-a_2}{b_1+b_2}, \end{aligned}$$

132 $$\begin{aligned} \kappa _2^{(B)}&= \frac{a_1-b_1\kappa _1^{(B)}}{b_1+b_2}, \end{aligned}$$

133 $$\begin{aligned} \kappa _3^{(B)}&= \frac{b_2-b_1}{b_1+b_2} \kappa _2^{(B)}. \end{aligned}$$

In the quoted paper, these three approximations are denoted by the sign $\sim$ instead of $\approx$. This would indicate that the approximations have the desirable property of being asymptotic under the condition that some identified variable becomes large. However, no such variable appears in the derivations. Because of this, the implicit claim by Bartlett et al. (1960) that the approximations are asymptotic has not been proved by them. It is, however, easy to use the results that we have derived to show that the BGL approximations of the first three cumulants are asymptotic. All one has to do is to use the reparameterization in (6)–(9). This leads to the following results:

134 $$\begin{aligned} \kappa _1^{(B)}&= x_1 N + x_2, \end{aligned}$$

135 $$\begin{aligned} \kappa _2^{(B)}&= y_1 N, \end{aligned}$$

136 $$\begin{aligned} \kappa _3^{(B)}&= z_1 N, \end{aligned}$$

where $x_1$, $x_2$, $y_1$, $z_1$ are given in (105), (106), (108), (110). This shows that the approximation (131) of the first cumulant of the QSD is equal to the sum of the first two terms of its asymptotic approximation for large *N*-values, while the approximations (132) and (133) of the second and third cumulants of he QSD are equal to the first terms of the corresponding asymptotic approximations. All three approximations are therefore asymptotic for large *N*. We note, however, that the BGL method does not by itself allow this important conclusion. Additional information about properties of the method will be uncovered in our study below of published results number three and four, referred to as BR1 and BR2.

The second published result that we comment on was presented by Matis and Kiffe (1996) in case $s=1$, and by Matis et al. (1998) when *s* is a positive integer. A valuable contribution of these papers is that they show that a system of ordinary differential equations (ODEs) for the first few cumulants of the unconditioned random variable *X*(*t*) can be derived from the PDE for the cumulant generating function $K(\theta ,t)$ of *X*(*t*). As already mentioned, we use a slight variation of this approach for deriving ODEs of the first few cumulants of the conditioned random variable $X^Q(t)$. It turns out that the first system of ODEs (for the cumulants of *X*(*t*)) serves as an approximation of the second system of ODEs (for the cumulants of $X^Q(t)$) under the condition that $R_0>1$. The critical points of the two systems of ODEs correspond to stationary distributions of the two random variables *X*(*t*) and $X^Q(t)$, respectively. The two systems of ODEs for the cumulants are not closed, in the sense that the number of cumulants is larger than the number of equations. This may appear as an undesirable property of the problem. Closure is clearly necessary if one wants or needs exact solutions. However, for our purposes it is important to realize that closure is not needed to find approximate solutions. To achieve closure, Matis et al. (1996, 1998) assume that all cumulants of sufficiently large order are equal to zero. This assumption is clearly at odds with our finding that all cumulants of the QSD are of order *O*(*N*). It turns out, however, that it leads to good numerical approximations of the cumulants. This behavior can be understood from our results. We note for example from Eqs. (36)–(38) that the right-hand sides of the ODEs for the first three cumulants when $s=4$ depend upon all seven cumulants $\kappa _1-\kappa _7$. But we note also in this case from (93)–(98) that our assumption that the cumulants $\kappa _4-\kappa _7$ are all *O*(*N*) leads us to conclude that they have no influence on the first three terms of the asymptotic approximation of $\kappa _1$, nor on the first two terms of the asymptotic approximation of $\kappa _2$, or on the first term of the asymptotic approximation of $\kappa _3$. The same conclusion can be drawn from the assumption made by Matis and Kiffe that the cumulants $\kappa _k$ are equal to zero for $k\ge 4$. This argument shows that cumulant approximations based on cumulant closure and an assumption of cumulant neglect can lead to asymptotic approximations if they are acceptable at all, as shown numerically in several cases by Matis et al. (1998). But the assumption that some cumulant $\kappa _k$ equals zero is incorrect and can lead to errors if one studies, e.g., the QSD instead of its cumulants.

The remaining three published results that we discuss here are all based on the BGL method. Renshaw (2011) has used the BGL method in an effort to improve the results by Bartlett et al. (1960) by giving additional terms in the approximations of the first three cumulants of the QSD. He proceeds to formulate two approaches that we refer to as BR1 and BR2, respectively, where the letters B and R are used to refer to Bartlett and Renshaw. In each of these two approaches, he proceeds to derive three relations between cumulants that turn out to be similar to relations between critical points of the system of ODEs for the first three cumulants. To describe his findings, we quote first from Renshaw (2011) the ODEs for the first three cumulants in the case $s=1$. They are derived from the PDE for the cumulant generating function and do not involve any approximations. For consistency in notation, we denote the time derivatives of the first three cumulants by $A^{(1)}$, $B^{(1)}$, and $C^{(1)}$, respectively, when $s=1$. We get

137 $$\begin{aligned} \kappa _1'&= A^{(1)} =a \kappa _1 - b (\kappa _1^2+\kappa _2), \end{aligned}$$

138 $$\begin{aligned} \kappa _2'&= B^{(1)} = 2 a \kappa _2 - b(4\kappa _1 \kappa _2 + 2\kappa _3) + c \kappa _1 - d(\kappa _1^2 +\kappa _2), \end{aligned}$$

139 $$\begin{aligned} \kappa _3'&= C^{(1)} = a (\kappa _1+3\kappa _3) - b(\kappa _1^2 + 6 \kappa _1\kappa _3 + 6 \kappa _2^2 + \kappa _2 + 3 \kappa _4) \nonumber \\&\quad + 3c \kappa _2 - d(6 \kappa _1 \kappa _2 + 3 \kappa _3). \end{aligned}$$

The same equations are found in Matis and Kiffe (1996), and, after using the reparameterization in (10)–(13), in Nåsell (2017).

The two approaches discussed by Renshaw (2011) are the third and fourth of the published results that we discuss. It turns out that the first two of the cumulant relations derived by Renshaw are in each of his two approaches equal to the expressions found by setting the cumulant derivatives $\kappa _1' = A^{(1)}$ and $\kappa _2' = B^{(1)}$ quoted above equal to zero. However, the third cumulant relation that he derives is different from what is found by setting $\kappa _3' = C^{(1)}$ equal to zero in each of the two approaches. In the approach BR1 the third cumulant relation is given by relation (3.5.21) in Renshaw’s book, while it is given by the expression following (3.5.38) in the same book in the approach BR2. We use the notations $C^{(a)}=0$ and $C^{(b)}=0$ to refer to these relations. For the first one of these, we find that $C^{(a)}$ is written as follows after using the reparameterization in (10)–(13):

140 $$\begin{aligned} C^{(a)}= & {} \mu (R_0-1) (\kappa _1^3 + 3 \kappa _1 \kappa _2 + \kappa _3) + \mu (R_0+1) \kappa _1^2 -\mu \frac{R_0-\alpha }{N} \kappa _1^3 \nonumber \\&- \mu \frac{R_0+\alpha }{N} (\kappa _1^4 + 6 \kappa _1^2 \kappa _2 + 4 \kappa _1 \kappa _3 + 3 \kappa _2^2 + \kappa _4). \end{aligned}$$

We proceed to derive asymptotic approximations of the first three cumulants of the QSD for this case. As in Sect. 6, our basic assumption is that the first four cumulants have the following asymptotic behaviors:

141 $$\begin{aligned} \kappa _1&= x_1 N + x_2 + \frac{x_3}{N} + \text {O}\left( \frac{1}{N^2}\right) , \end{aligned}$$

142 $$\begin{aligned} \kappa _2&= y_1 N + y_2 + \text {O} \left( \frac{1}{N} \right) , \end{aligned}$$

143 $$\begin{aligned} \kappa _3&= z_1 N + \text {O}(1), \end{aligned}$$

144 $$\begin{aligned} \kappa _4&= u_1 N + \text {O}(1). \end{aligned}$$

By inserting these asymptotic approximations of the first four cumulants into the expressions for $A^{(1)}, B^{(1)}, C^{(a)}$ in (137), (138), (140), we find that asymptotic approximations for them can be written as follows:

145 $$\begin{aligned} A^{(1)}&= A_1^{(1)} N + A_2^{(1)} + \frac{A_3^{(1)}}{N} + \text {O}\left( \frac{1}{N^2} \right) , \end{aligned}$$

146 $$\begin{aligned} B^{(1)}&= B_1^{(1)} N + B_2^{(1)} + \text {O} \left( \frac{1}{N} \right) , \end{aligned}$$

147 $$\begin{aligned} C^{(a)}&= C^{(a)}_3 N^3 + C^{(a)}_2 N^2 + C^{(a)}_1 N + \text {O}(1), \end{aligned}$$

where

148 $$\begin{aligned} A_1^{(1)}&= \mu (R_0-1) x_1 -\mu (R_0+\alpha ) x_1^2, \end{aligned}$$

149 $$\begin{aligned} A_2^{(1)}&= \mu (R_0-1) x_2 - \mu (R_0+\alpha ) (2 x_1 x_2 + y_1), \end{aligned}$$

150 $$\begin{aligned} A_3^{(1)}&= \mu (R_0-1) x_3 - \mu (R_0+\alpha ) (2 x_1 x_3 + x_2^2 +y_2), \end{aligned}$$

151 $$\begin{aligned} B_1^{(1)}&= 2\mu (R_0-1) y_1 + \mu (R_0+1) x_1 -\mu (R_0-\alpha ) x_1^2 - 4\mu (R_0+\alpha ) x_1 y_1 , \end{aligned}$$

152 $$\begin{aligned} B_2^{(1)}&= 2\mu (R_0-1) y_2 + \mu (R_0+1) x_2 - \mu (R_0-\alpha ) (2 x_1 x_2 + y_1) \nonumber \\&\quad - \mu (R_0+\alpha ) (4 x_1 y_2 + 4 x_2 y_1+ 2 z_1), \end{aligned}$$

153 $$\begin{aligned} C^{(a)}_3&=\mu (R_0-1) x_1^3 -\mu (R_0+\alpha ) x_1^4, \end{aligned}$$

154 $$\begin{aligned} C^{(a)}_2&= \mu (R_0-1) (3 x_1^2 x_2 + 3 x_1 y_1) + \mu (R_0+1) x_1^2 - \mu (R_0-\alpha ) x_1^3\nonumber \\&\quad - \mu (R_0+\alpha ) (4 x_1^3 x_2 + 6 x_1^2 y_1), \end{aligned}$$

155 $$\begin{aligned} C^{(a)}_1&= \mu (R_0-1) (3 x_1^2 x_3 + 3 x_1 x_2^2 + 3 x_1 y_2 + 3 x_2 y_1 + z_1)\nonumber \\&\quad + 2 \mu (R_0+1) x_1 x_2 - 3 \mu (R_0-\alpha ) x_1^2 x_2 \nonumber \\&\quad - \mu (R_0 + \alpha ) (4 x_1^3 x_3 + 6 x_1^2 x_2^2 + 6 x_1^2 y_2 + 12 x_1 x_2 y_1 + 4 x_1 z_1 + 3 y_1^2). \end{aligned}$$

The basic mathematical problem at this point is to determine the six coefficients $x_1, x_2, x_3, y_1, y_2, z_1$ so that the following three conditions are satisfied:

156 $$\begin{aligned}&A^{(1)} = \text {O} \left( \frac{1}{N^2} \right) , \end{aligned}$$

157 $$\begin{aligned}&B^{(1)} = \text {O} \left( \frac{1}{N} \right) , \end{aligned}$$

158 $$\begin{aligned}&C^{(a)} = \text {O} \left( 1 \right) . \end{aligned}$$

These conditions are satisfied by setting the eight expressions (148)–(155) equal to zero. We proceed to solve the resulting eight equations for the six unknown coefficients $x_1$, $x_2$, $x_3$, $y_1$, $y_2$, $z_1$. At this point it appears that there are more equations than unknowns, but the apparent problem that this causes will readily be solved. The equation $A_1^{(1)}=0$ is first solved for $x_1$, and the spurious solution $x_1=0$ is excluded. As above, as soon as the solution is found for any of the coefficients $x_1$, $x_2$, $x_3$, $y_1$, $y_2$, $z_1$, it is inserted into the remaining unsolved equations. The equation $B_1^{(1)}=0$ is then solved for $y_1$, and the equation $A_2^{(1)}=0$ is solved for $x_2$. After this we find that the two expressions $C^{(a)}_3$ and $C^{(a)}_2$ can both be determined from these values, and that they both are equal to zero. Thus, the number of equations is reduced to be equal to the number of unknown coefficients. In the last steps we use the equation $A_3^{(1)}=0$ to express $x_3$ as a function of $y_2$, the equation $B_2^{(1)}=0$ to solve for $z_1$ as a function of $y_2$, and finally the equation $C_1^{(a)}=0$ to solve for $y_2$. The result of these evaluations is that the six coefficients $x_1$, $x_2$, $x_3$, $y_1$, $y_2$, $z_1$ can be expressed as follows as functions of the model parameters $R_0$ and $\alpha$:

159 $$\begin{aligned} x_1&= \frac{R_0-1}{R_0+\alpha }, \end{aligned}$$

160 $$\begin{aligned} x_2&= - \frac{(\alpha +1)R_0}{(R_0+\alpha )(R_0-1)}, \end{aligned}$$

161 $$\begin{aligned} x_3&= \frac{(\alpha +1) R_0 (R_0^2+\alpha - 5 (\alpha +1) R_0)}{(R_0+\alpha ) (R_0-1)^3}, \end{aligned}$$

162 $$\begin{aligned} y_1&= \frac{(\alpha +1) R_0}{(R_0+\alpha )^2}, \end{aligned}$$

163 $$\begin{aligned} y_2&= - \frac{(\alpha +1) R_0 (R_0^2 + \alpha - 4 (\alpha +1) R_0) }{(R_0+\alpha )^2 (R_0-1)^2 }, \end{aligned}$$

164 $$\begin{aligned} z_1&= \frac{(\alpha +1) R_0 (R_0^2+\alpha - 3 (\alpha +1) R_0) }{(R_0+\alpha )^3 (R_0-1) }. \end{aligned}$$

Comparisons with (105)–(110) show that the expressions for the three coefficients $x_1$, $x_2$, and $y_1$ are equal to the corresponding expressions derived in Sect. 7 using our preferred method where asymptotic approximations of the cumulants are derived from the ODEs for the cumulants without attempting to close the equations. The comparisons also show that the expressions for the remaining three coefficients $x_3$, $y_2$, and $z_1$ disagree between our approach and BR1. This means that BR1 does not give any improvement on the approximations of the first two cumulants established by Bartlett et al. (1960). The reason for this is that one or more of the various approximation steps that are taken in using the BGL method brings in errors. Renshaw (2011) appears to share this view in his brief discussion of this issue after his formula (3.5.21). We conclude that the BGL method brings in errors of unknown magnitude, and therefore is unsuitable for deriving approximations of the cumulants of the QSD beyond those derived by Bartlett et al. (1960). Support for this claim is given by the numerical evaluations reported in Table 5. They indicate that the error terms of the approximations of the cumulants $\kappa _1, \kappa _2, \kappa _3$, derived in Nåsell (2017), are of the orders O(1/$N^2$), O(1/*N*), O(1), respectively, just as expected. But we find also that the error terms of the approximations derived via the BR1 approach are larger than this by an order of magnitude. The BR1 approximation of $\kappa _3$ in this case is useless, since the error terms actually grow with *N*.

**Table 5:** Numerical evaluations of the error terms of three different approximations of the first three cumulants of the QSD of the stochastic Verhulst logistic model

Approx	Cumulant	$$N=100 $$	$$N=200$$	$$N=400$$
N	$\kappa _1$	$-\,697*10^{-7}$	$-\,171*10^{-7}$	$-\,42*10^{-7}$
N	$\kappa _2$	$445*10^{-5}$	$219*10^{-5}$	$108*10^{-5}$
N	$\kappa _3$	$-\,226*10^{-3}$	$-\,222*10^{-3}$	$-\,220*10^{-3}$
BR1	$\kappa _1$	$-\,311*10^{-5}$	$-\,154*10^{-5}$	$-\,76*10^{-5}$
BR1	$$\kappa _2$$	$$253*10^{-3} $$	$$251*10^{-3} $$	$$250*10^{-3}$$
BR1	$\kappa _3$	$-$ 21	$-$ 41	$-$ 82
BB	$\kappa _1$	$-\,359*10^{-5}$	$-\,178*10^{-5}$	$-\,88*10^{-5}$
BB	$\kappa _2$	$292*10^{-3}$	$290*10^{-3}$	$289*10^{-3}$
BB	$\kappa _3$	$-\,10.2$	$-\,20.3$	$-\,40.3$

Results are shown for $R_0=10$, $\alpha =1$, $s=1$, and the *N*-values 100, 200, and 400. Approximation N uses the results presented in this paper, while Approximation BR1 uses the first version of the Bartlett–Renshaw approach, and Approximation BB is taken from Sect. 4 of the paper by Bhowmick et al. The table indicates that the error terms of $\kappa _1, \kappa _2, \kappa _3$ in Approximation *N* are reduced by approximately 4, 2, and 1, respectively, for each doubling of *N*, while in approximations BR1 and BB the error terms of $\kappa _1$ and $\kappa _2$ are reduced by approximately 2 and 1, respectively, for each doubling of *N*, and the error terms of $\kappa _3$ are seen to grow with *N*. The derivations of the numerical results here are documented in Nåsell (2018)

We turn now to a consideration of the second version BR2 of the Bartlett–Renshaw approach. Renshaw (2011) uses it to derive a new expression for the third cumulant relation. It is given after relation (3.5.38) in his book. After writing $\mu _4 = \kappa _4 + 3 \kappa _2^2$ and using the reparameterization (10)–(13), it can be written $C^{(b)}=0$, where $C^{(b)}$ is as follows:

165 $$\begin{aligned} C^{(b)}= & {} \mu (R_0-1) (\kappa _1 \kappa _2 + \kappa _3) + \mu (R_0+1) \kappa _2 - \mu \frac{R_0-\alpha }{N} (2 \kappa _1 \kappa _2 + \kappa _3) \nonumber \\&- \mu \frac{R_0+\alpha }{N} (\kappa _1^2 \kappa _2 + 2 \kappa _1 \kappa _3 + 3 \kappa _2^2 + \kappa _4). \end{aligned}$$

We proceed to derive asymptotic approximations of the first three cumulants of the QSD for this case, using the three cumulant relations $A^{(1)}=0$, $B^{(1)}=0$, and $C^{(b)}=0$. As above, we assume that the first four cumulants have the following asymptotic behaviors:

166 $$\begin{aligned} \kappa _1&= x_1 N + x_2 + \frac{x_3}{N} + \text {O}\left( \frac{1}{N^2} \right) , \end{aligned}$$

167 $$\begin{aligned} \kappa _2&= y_1 N + y_2 + \text {O}\left( \frac{1}{N} \right) , \end{aligned}$$

168 $$\begin{aligned} \kappa _3&= z_1 N + \text {O}(1), \end{aligned}$$

169 $$\begin{aligned} \kappa _4&= u_1 N + \text {O}(1). \end{aligned}$$

By inserting these asymptotic approximations of the first four cumulants into the expressions for $A^{(1)}$, $B^{(1)}$, and $C^{(b)}$, we find that asymptotic approximations for them can be written as follows:

170 $$\begin{aligned}&A^{(1)} = A_1^{(1)} N + A_2^{(1)} + \frac{A_3^{(1)}}{N} + \text {O}\left( \frac{1}{N^2} \right) , \end{aligned}$$

171 $$\begin{aligned}&B^{(1)} = B_1^{(1)} N + B_2^{(1)} + \text {O}\left( \frac{1}{N} \right) , \end{aligned}$$

172 $$\begin{aligned}&C^{(b)} = C^{(b)}_2 N^2 + C^{(b)}_1 N + \text {O}(1). \end{aligned}$$

Expressions for the five quantities $A_1^{(1)}, A_2^{(1)}, A_3^{(1)}, B_1^{(1)}, B_2^{(1)}$ are given in (148)–(152), while $C^{(b)}_2$ and $C^{(b)}_1$ are equal to

173 $$\begin{aligned} C^{(b)}_2&= \mu (R_0-1) x_1 y_1 - \mu (R_0+\alpha ) x_1^2 y_1, \end{aligned}$$

174 $$\begin{aligned} C^{(b)}_1&= \mu (R_0-1) (x_1 y_2 + x_2 y_1 + z_1) + \mu (R_0+1) y_1 - 2 \mu (R_0 - \alpha ) x_1 y_1 \nonumber \\&\quad - \mu (R_0+\alpha ) \left( x_1^2 y_2 + 2 x_1 x_2 y_1 + 2 x_1 z_1 + 3 y_1^2\right) . \end{aligned}$$

We set these seven expressions equal to zero, and solve the resulting seven equations for the six unknown coefficients $x_1, x_2, x_3, y_1, y_2, z_1$. The value of $x_1$ is found by solving the equation $A_1^{(1)}=0$ and excluding the spurious solution $x_1=0$. The values of $y_1$ and $x_2$ are then found by first solving the equation $B_1^{(1)}=0$ for $y_1$, and then solving the equation $A_2^{(1)}=0$ for $x_2$. The values of $x_1$, $x_2$, and $y_1$ are the same as the ones found in (159), (160), (162). Furthermore, we insert these values of $x_1, x_2, y_1$ into (173) and (174). This shows that $C^{(b)}_2=0$ and that therefore the number of equations available for solving for the six coefficients is reduced from seven to six. We also get

175 $$\begin{aligned} C^{(b)}_1 = -(R_0-1) z_1 - \frac{(\alpha +1) R_0 (R_0-\alpha ) (R_0-1)}{(R_0+\alpha )^3}. \end{aligned}$$

To determine the three coefficients $x_3$, $y_2$, $z_1$ we solve the equation $C^{(b)}_1=0$ for $z_1$, the equation $B_2^{(1)}=0$ for $y_2$, and the equation $A_3^{(1)}=0$ for $x_3$. The rather surprising result is that the values of the six coefficients $x_1$, $x_2$, $x_3$, $y_1$, $y_2$, $z_1$ are found to be the same as in (105)–(110). This means that even though the explicit results from the BR2 method are different from the results from our preferred method, the asymptotic approximations agree.

In his development of BR2, Renshaw (2011) uses the equation $C^{(b)}_1=0$ to derive the relation

176 $$\begin{aligned} \kappa _3 \approx -\frac{R_0-\alpha }{R_0+\alpha } \kappa _2. \end{aligned}$$

By using the reparameterization in (11) and (13) we find that this relation was shown to hold already by Bartlett et al. (1960). It is seen from (108) and (110) that this relation is asymptotic for large *N*. However, the arguments used by Renshaw in his derivation can be criticized. As also pointed out by Bhowmick et al. (2016), it does not make sense to assume that $\kappa _1$ is large in comparison with $a_1, b_1, a_2, b_2$, since these four parameters are rates whose values depend on the unit of time, which is arbitrary, while $\kappa _1$ is independent of the time unit. It is also unrealistic to assume that $\kappa _2$ is small compared with $\kappa _1$, since it contradicts our finding that all cumulants are *O*(*N*).

Renshaw’s two efforts to extend the BGL approximation beyond what was derived by Bartlett et al. (1960) leads to two different conclusions. In one case (BR2) the results provide improvements, while they do not in the other case (BR1). This is enough to conclude that the BGL method cannot be trusted to give improved results without further investigations.

We turn now to consider the results reported by Bhowmick et al. (2016). Actually, they study a more general model than the one that we are concerned with here. The population birth rate $\lambda _n$ in their model is given by

177 $$\begin{aligned} \lambda _n = \mu R_0 \left( 1 - \alpha _1 \left( \frac{n}{N}\right) ^\beta \right) n^\delta , \end{aligned}$$

while their population death rate $\mu _n$ equals

178 $$\begin{aligned} \mu _n = \mu \left( 1 + \alpha _2 \left( \frac{n}{N}\right) ^\beta \right) n^\delta . \end{aligned}$$

They allow $\delta$ to be a positive number, not necessarily an integer. This leads to difficulties in determining dimensions and biological interpretations of the parameters whenever $\delta \ne 1$. To avoid such difficulties, we limit ourselves to a study of their model in the special case when $\delta =1$. To agree with our model formulation we furthermore put $\beta =s=1$, $\alpha _1=1$ and $\alpha _2=\alpha$.

By using results derived by Bhowmick et al. (2016) and reported in Sect. 4 of their paper, we find that their approximation $\kappa _1^{(BB)}$ of the first cumulant $\kappa _1$ equals

179 $$\begin{aligned} \kappa _1^{(BB)} = \frac{R_0-1}{R_0+\alpha } N \frac{1}{1 + H/N}, \end{aligned}$$

where

180 $$\begin{aligned} H = \frac{(\alpha +1) R_0}{(R_0-1)^2}. \end{aligned}$$

By including three terms in the asymptotic approximation of $\kappa _1$ in (179), we find that

181 $$\begin{aligned} \kappa _1^{(BB)} = \frac{R_0-1}{R_0+\alpha } N - \frac{(\alpha +1)R_0}{(R_0+\alpha )(R_0-1) } + \frac{(\alpha +1)^2 R_0^2}{(R_0+\alpha )(R_0-1)^3} \frac{1}{N} + \text {O} \left( \frac{1}{N^2} \right) .\nonumber \\ \end{aligned}$$

From relation (25) in Bhowmick et al., we find that their approximation $\kappa _2^{(BB)}$ of the variance $\kappa _2$ equals

182 $$\begin{aligned} \kappa _2^{(BB)} = {\kappa _2^{(BB)}}^2 \frac{H}{N}. \end{aligned}$$

By including two terms in the asymptotic approximation of $\kappa _2^{(BB)}$, we find that

183 $$\begin{aligned} \kappa _2^{(BB)} = \frac{(\alpha +1) R_0}{(R_0+\alpha )^2}N - \frac{2(\alpha +1)^2 R_0^2}{(R_0+\alpha )^2 (R_0-1)^2} + \text {O}\left( \frac{1}{N} \right) . \end{aligned}$$

Bhowmick et al. use four quantities *A*, *B*, *C*, and *D* in a formula that determines their approximation $\kappa _3^{(BB)}$ of the third cumulant $\kappa _3$ as follows:

184 $$\begin{aligned} \kappa _3^{(BB)} = - \frac{A+B}{C+D} \kappa _2^{(BB)}. \end{aligned}$$

These four quantities are defined in terms of the parameters that are used to describe the first of the two model formulations in Sect. 2. After reparameterization they can be expressed as follows:

185 $$\begin{aligned}&A = a - b \kappa _1^{(BB)} = \mu (R_0-1) - \mu \frac{R_0+\alpha }{N} \kappa _1^{(BB)}, \end{aligned}$$

186 $$\begin{aligned}&B = \frac{c}{\kappa _1^{(BB)}} - 2d = \mu \frac{R_0+1}{\kappa _1^{(BB)}} - 2\mu \frac{R_0-\alpha }{N}, \end{aligned}$$

187 $$\begin{aligned}&C = \frac{a}{\kappa _1^{(BB)}} - 2b = \mu \frac{R_0-1}{\kappa _1^{(BB)}} - 2\mu \frac{R_0+\alpha }{N}, \end{aligned}$$

188 $$\begin{aligned}&D = - \frac{d}{\kappa _1^{(BB)}} = - \mu \frac{R_0-\alpha }{N \kappa _1^{(BB)}}. \end{aligned}$$

Thus, all four of these quantities are determined as functions of $\kappa _1^{(BB)}$. We use the asymptotic approximation of $\kappa _1^{(BB)}$ in (181) to determine one-term asymptotic approximations of each of *A*, *B*, *C*, *D*. Two terms of the asymptotic approximation of $\kappa _1^{(BB)}$ are needed for *A*, while one term suffices for the remaining three quantities. The results are

189 $$\begin{aligned}&A = \mu \frac{(\alpha +1)R_0}{R_0-1} \frac{1}{N}+ \text {O}\left( \frac{1}{N^2} \right) , \end{aligned}$$

190 $$\begin{aligned}&B = -\mu \frac{R_0^2+\alpha - 3(\alpha +1)R_0}{R_0-1} \frac{1}{N} +\text {O} \left( \frac{1}{N^2} \right) , \end{aligned}$$

191 $$\begin{aligned}&C = - \mu (R_0+\alpha )\frac{1}{N} +\text {O} \left( \frac{1}{N^2} \right) , \end{aligned}$$

192 $$\begin{aligned}&D = -\mu \frac{R_0^2-\alpha ^2}{R_0-1} \frac{1}{N^2} + \text {O} \left( \frac{1}{N^3} \right) . \end{aligned}$$

It follows from this that the one-term asymptotic approximation of the ratio $(A+B)/(C+D)$ equals

193 $$\begin{aligned} \frac{A+B}{C+D} = \frac{R_0^2+\alpha - 4(\alpha +1) R_0}{(R_0+\alpha ) (R_0-1)} + \text {O}\left( \frac{1}{N} \right) . \end{aligned}$$

By inserting this result and the one-term asymptotic approximation of $\kappa _2^{(BB)}$ from (183) into (184), we get

194 $$\begin{aligned} \kappa _3^{(BB)} = - \frac{(\alpha +1) R_0 (R_0^2 + \alpha - 4 (\alpha +1)R_0)}{(R_0+\alpha )^3 (R_0-1)} N + \text {O}(1). \end{aligned}$$

The resulting asymptotic approximations of the first three cumulants derived by Bhowmick et al. are found in (181), (183), and (194). These can be compared with the results derived by using our preferred method and found in (105)–(110). The comparisons show that the first two terms of the asymptotic approximation of $\kappa _1^{(BB)}$ agree with the corresponding terms of the asymptotic approximation of the true first cumulant $\kappa _1$, but there is disagreement between the third terms. Moreover, the first term of the asymptotic approximation of $\kappa _2^{(BB)}$ agrees with the first term of the asymptotic approximation of the true second cumulant $\kappa _2$, but there is disagreement between the second terms. Finally, there is disagreement between the one-term asymptotic approximation of $\kappa _3^{(BB)}$ and the corresponding one-term asymptotic approximation of the true third cumulant $\kappa _3$ . Independent supports for these conclusions are found from the numerical evaluations of the error terms of the results presented by Bhowmick et al. and given in Table 5. We see here that the error term for the BB approximation of $\kappa _1$ is reduced by the factor two for each doubling of *N*. This is indicative of an error that is of magnitude of $\text {O}(1/N)$. In addition, the error term for the BB approximation of $\kappa _2$ is found to be approximately independent of *N*, which indicates that it is of the magnitude of $\text {O}(1)$. The error term for the BB approximation of $\kappa _3$ is, finally, found to be approximately proportional to *N*, which makes the corresponding approximation useless. The reason for the larger errors in the results arrived at by Bhowmick et al. is that one or more of the approximation steps taken in the application of BGL method brings in errors. This is similar to the reason for the large errors of the BR1 result, commented on above.

It is interesting to note that the study by Bhowmick et al. (2016) is the only one among the five studies commented on in this section that uses the parameterization that accompanies the second of the two model formulations described in Sect. 2. However, we note also that Bhowmick et al. do not make use of the important property that this model formulation offers, namely the availability of the large parameter *N* that can be used for deriving asymptotic approximations.

## Concluding Comments

The method that we have used here to derive asymptotic approximations of the first few cumulants of the QSD of the stochastic power law logistic model emphasizes the importance of the second model formulation in Sect. 2. It gives access to two parameters that are basic for our study, namely the maximum population size *N* and the threshold parameter $R_0$. We have shown that the condition $R_0>1$ for large *N* is required for the results that we have presented here, while both the moment closure method and the three approaches BR1, BR2, and BB based on the BGL method have the serious weaknesses that they cannot produce conditions for validity of the approximations that are derived. In addition, we note that magnitudes of approximation errors are easy to establish in our method, as they are for any asymptotic approximation, while they can not be produced in the moment closure method, nor in approaches based on the BGL method. We note furthermore that spurious solutions that could require large efforts to eliminate appeared in early studies based on moment closure, while the spurious solutions that appear in the method that we use here are easy to identify and eliminate. Our results show that the number of spurious solutions is equal to the parameter *s* whenever *s* is a positive integer. As a further comment, we note that the dependence of the new approximations on the model parameters is explicit in our approach, while they are unknown in results based on moment closure and in BR1 and BR2.

We have found that our method based on the second model formulation of Sect. 2, and followed by a search for asymptotic approximations, provides a powerful approach for determination of approximations of the first few cumulants of the QSD for the power law logistic model. We have also shown that the method of determining asymptotic approximations can be used to study other approaches to the same problem.

We conclude that the method of determining ODEs for the first few cumulants of the QSD introduced by Matis and Kiffe (1996) is preferred over the BGL method for deriving relations between the cumulants. An important reason for this is that the ODEs for the first few cumulants of the QSD are exact, while the BGL method can cause errors of unknown magnitude. We emphasize the obvious fact that whenever exact solutions of a mathematical problem are difficult to establish, then one should search for approximations. Furthermore, approximation methods are then definitely preferred that give both conditions for validity of the approximations and magnitudes of approximation errors. Because of this, we conclude that our method for determining asymptotic approximations of the first few cumulants is preferred over any other method that has been used on this problem.
